# How do stakeholders experience the adoption of electronic prescribing systems in hospitals? A systematic review and thematic synthesis of qualitative studies

**DOI:** 10.1136/bmjqs-2018-009082

**Published:** 2019-07-29

**Authors:** Albert Farre, Gemma Heath, Karen Shaw, Danai Bem, Carole Cummins

**Affiliations:** 1 School of Nursing and Health Sciences, University of Dundee, Dundee, UK; 2 Life and Health Sciences, Aston University, Birmingham, UK; 3 Institute of Applied Health Research, University of Birmingham, Birmingham, UK

**Keywords:** electronic prescribing, computerisedprovider order entry (cpoe, systematic review, qualitative research, thematic synthesis

## Abstract

**Background:**

Electronic prescribing (ePrescribing) or computerised provider/physician order entry (CPOE) systems can improve the quality and safety of health services, but the translation of this into reduced harm for patients remains unclear. This review aimed to synthesise primary qualitative research relating to how stakeholders experience the adoption of ePrescribing/CPOE systems in hospitals, to help better understand why and how healthcare organisations have not yet realised the full potential of such systems and to inform future implementations and research.

**Methods:**

We systematically searched 10 bibliographic databases and additional sources for citation searching and grey literature, with no restriction on date or publication language. Qualitative studies exploring the perspectives/experiences of stakeholders with the implementation, management, use and/or optimisation of ePrescribing/CPOE systems in hospitals were included. Quality assessment combined criteria from the Critical Appraisal Skills Programme Qualitative Checklist and the Standards for Reporting Qualitative Research guidelines. Data were synthesised thematically.

**Results:**

79 articles were included. Stakeholders’ perspectives reflected a mixed set of positive and negative implications of engaging in ePrescribing/CPOE as part of their work. These were underpinned by further-reaching change processes. Impacts reported were largely practice related rather than at the organisational level. Factors affecting the implementation process and actions undertaken prior to implementation were perceived as important in understanding ePrescribing/CPOE adoption and impact.

**Conclusions:**

Implementing organisations and teams should consider the breadth and depth of changes that ePrescribing/CPOE adoption can trigger rather than focus on discrete benefits/problems and favour implementation strategies that: consider the preimplementation context, are responsive to (and transparent about) organisational and stakeholder needs and agendas and which can be sustained effectively over time as implementations develop and gradually transition to routine use and system optimisation.

## Introduction

The medication use process in hospital settings is generally understood to comprise four interrelated stages: prescribing, dispensing, administering and monitoring.^1^ In practice, these involve a broad range of health professionals, documents, practices, situations, settings and multiple inter-related processes, the interplay of which can give rise to several risks and errors with the potential to result in patient harm.^2–5^ Electronic prescribing (ePrescribing) or computerised provider/physician order entry (CPOE) systems can improve patient safety and the quality of health services by reducing risks associated with medication errors^6–8^ and by improving organisational efficiency and health professionals’ performance throughout the medication process.^7 9^

However, the translation of such improvements into reduced harm for patients is still unclear.^8 10^ It has become increasingly clear that the implementation of ePrescribing/CPOE systems may create unintended consequences and introduce new safety issues once in use.^9 11–15^ In this context, in light of the inherent complexity of the medication process and the difficulty of examining it in isolation from other interrelated processes and contextual factors, a growing body of qualitative research has provided insights into key implementation and use issues concerning ePrescribing/CPOE systems in hospital settings^9 16–23^ for nearly two decades.^24^ An interpretative examination of such body of qualitative evidence would enable better understanding of why and how healthcare organisations have not yet realised the full potential of such systems and would inform future implementations and research. With this motivation, we conducted a systematic review of qualitative studies addressing the following question: how do stakeholders experience the adoption of ePrescribing/CPOE systems in hospitals?

We aimed to identify, collate, assess and synthesise primary qualitative research relating to the perceptions and experiences of those involved in, or affected by, the implementation, management, use and/or optimisation of ePrescribing/CPOE systems in hospital settings.^25^

The review has three important elements absent from previous attempts to synthesise primary qualitative research on this topic^26^: (1) it employs an interpretative (rather than aggregative) analytical approach; (2) it draws on all reported stakeholders’ perspectives, not just those of health professionals; and (3) it includes research at any stage of ePrescribing/CPOE adoption, from implementation through to routine use.

This is of particular importance given the complex, sociotechnical nature of ePrescribing/CPOE systems and the inherent difficulty of establishing the end-point of the implementation process. Implementation is generally understood as the transitional period or set of activities between the organisational decision to adopt an intervention and the point at which it becomes assimilated as routine use.^27–29^ This separation is not straightforward for interventions with such complexity: this judgement is multifaceted, highly contingent on multiple perspectives and context dependent. Overlaps between system implementation, routine use and system optimisation issues across settings and organisations are integral in the literature. Therefore, an interpretative examination of ePrescribing/CPOE across studies is important in that it enables incorporation of multiple perspectives and accommodation of a wide range of conceptualisations about the implementation process, in a holistic analytical approach.

## Methods

We registered and published a peer-reviewed protocol^25^ following ENTREQ guideline recommendations,^30^ adopting systematic search methodology and thematic synthesis.^31^

### Search strategy

The following bibliographic databases were searched from inception to October 2018: MEDLINE, MEDLINE In Process, Embase, PsycINFO, Social Policy and Practice, CINAHL, The Cochrane Library (CDSR, DARE and CENTRAL databases), Nursing and Allied Health Sources, Applied Social Sciences Index and Abstracts and Scopus. Additional sources were Sciences and Social Sciences Citation Index and grey literature (Healthcare Management Information Consortium, Conference Proceedings Citation Index and Sociological Abstracts). Citations in relevant reviews and included studies were checked. Selected specialist journals were hand searched.

A comprehensive search strategy was developed, employing a combination of search filters,^32 33^ text words and index terms relating to qualitative research and relevant interventions, including variations and permutations used in similar reviews^7 26 34–36^ with no restriction on date or language. The sample strategy in online supplementary appendix 1 was adapted for each bibliographic database.

10.1136/bmjqs-2018-009082.supp1 Supplementary data

### Inclusion criteria

Qualitative studies (standalone or within mixed-methods designs) exploring stakeholder perspectives/experiences of implementation, management, use and/or optimisation of ePrescribing/CPOE systems in hospitals were included. Any electronic system or subsystem involving the prescription and/or administration phase of the medication process were included. Electronic systems involving other phases of the medication process (eg, systems for stock control) but not prescribing were excluded. Where CPOE systems allowed the ordering of anything other than medication, studies were excluded unless findings specific to medication were reported separately. Any types of participants/perspectives (eg, doctors, nurses, managers, service users and IT staff) were eligible. Eligible settings included hospital-based care settings (eg, wards, clinics, areas, specialities or whole organisations). All articles were independently screened by two reviewers. Discrepancies were resolved by discussion until consensus was reached.

### Quality appraisal

Quality appraisal of included studies was conducted using a tool (online supplementary table 1) derived from the Critical Appraisal Skills Programme Qualitative Research Checklist^37^ and the Standards for Reporting Qualitative Research.^38^ The methodological quality of each study was independently appraised by two reviewers. Disagreements were resolved by discussion until consensus was reached. Acknowledging the inherent difficulty of appraising all aspects of quality of qualitative research,^39^ studies were not excluded based on the quality/adequacy of the reporting. Instead, the quality of studies was taken into consideration during data synthesis^40^ by exploring whether any particular finding or group of findings were dependent, either exclusively or disproportionately, on one or more studies classed as ‘low-quality’ or ‘inadequately reported’.

10.1136/bmjqs-2018-009082.supp2 Supplementary data

### Data extraction

Articles were read in full before data were extracted and recorded by two reviewers using a piloted data extraction form (online supplementary table 2). Study findings were all text and tables labelled as 'results' or 'findings' in each article including verbatim data extracts from participants and authors’ descriptions, summaries and interpretations of primary data. Extracted data were imported into NVivo V.11 to assist the coding, data management and data synthesis process.

10.1136/bmjqs-2018-009082.supp3 Supplementary data

### Data synthesis

Data were synthesised using a thematic synthesis approach^31^ with three overlapping interrelated stages: (1) line-by-line coding of the findings; (2) categorisation of codes into descriptive themes; and (3) development of analytical themes to describe and/or explain descriptive themes.

A multiple coding strategy was employed, with the lead author coding the whole dataset and the remaining review team coding subsets to ensure all data were independently double-coded. Regular meetings were held throughout the data synthesis process to carry out reviewer triangulation comparing reviewer codebooks, descriptive/emerging themes and interpretations until a coding framework was agreed. This was then applied to the whole dataset by the lead author and revised and refined with the team. Subsequent meetings focused on: categorisation of initial codes into descriptive themes; development, discussion and agreement of analytical themes and interpretative framework; and discussion, refinement and establishment of final synthesis findings.

## Results

**Figure 1:**
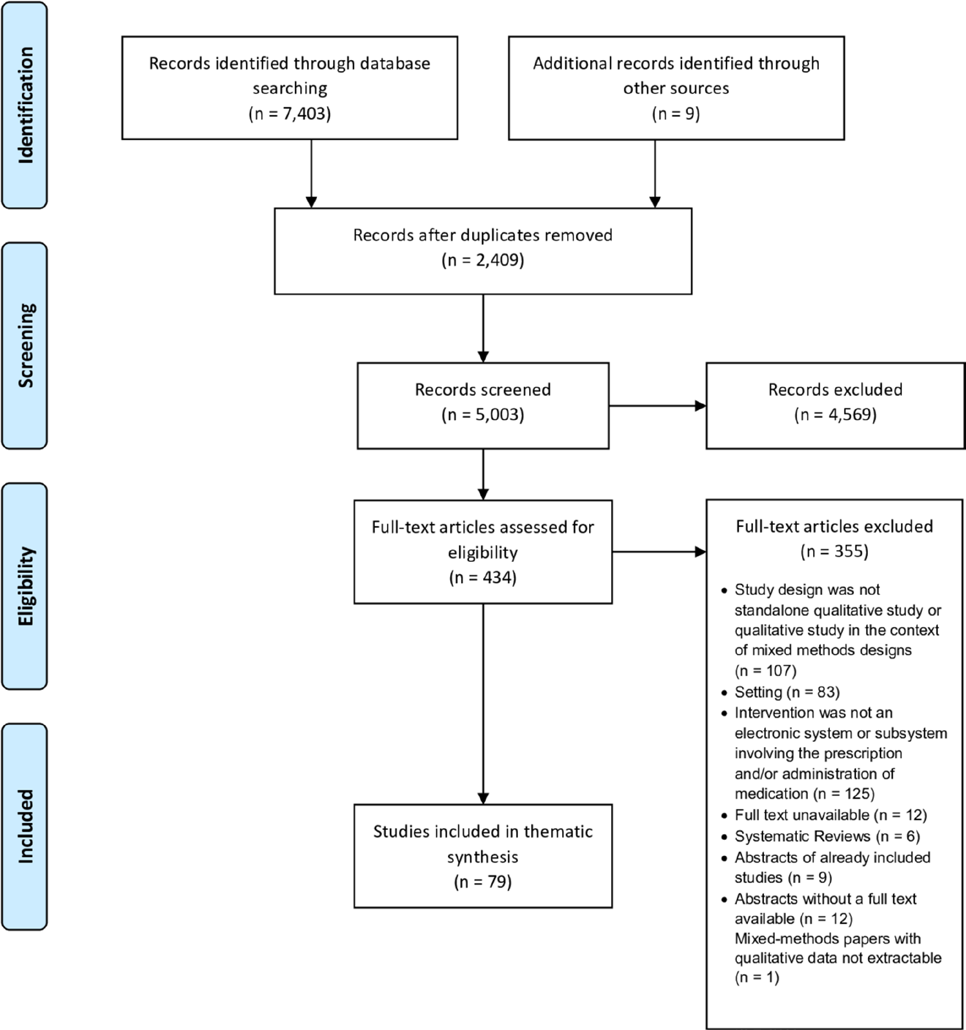
PRISMA flow diagram illustrating the study selection process. PRISMA, Preferred Reporting Items for Systematic Reviews and Meta-Analyses.

Systematic searches yielded 5003 records, which were assessed against the inclusion criteria. Abstract screening resulted in 434 records considered eligible or inconclusive. Full-text articles were then retrieved and assessed for eligibility, with 79 papers included in the final synthesis (figure 1). Included papers reported data from 15 countries (UK=26, USA=25, the Netherlands=9 and Australia=8). Study samples ranged from 10 to 1018 participants. Articles mainly focused on the perspectives of health professionals (in clinical, administrative, technical and leadership roles) with a few including other stakeholders (eg, patients, carers, policymakers or systems suppliers). Study settings included adult and paediatric acute care hospitals, general and community hospitals, medical and surgical wards and hospital-based clinics. Key characteristics of included studies are presented in online supplementary table 3.

10.1136/bmjqs-2018-009082.supp4 Supplementary data

Our quality assessment (table 1, online supplementary table 4) concluded that, overall, articles reported valuable research and credible findings. Nevertheless, 29 of the 79 papers did not report or employ any techniques to enhance trustworthiness (such as multiple coding or triangulation) in their data analysis process, adding to the inherent difficulty of appraising the credibility of findings. Only 10 of the 79 papers clearly and explicitly addressed the relationship between researchers and participants, with a further 16 papers providing only some relevant information, adding to the difficulty of evaluating the impact of these aspects on study findings.

10.1136/bmjqs-2018-009082.supp5 Supplementary data

**Table 1:** Summary of quality assessment of included studies (n=79)

	Yes, n (%)	Partially, n (%)	No, n (%)	Unclear, n (%)
Was the research problem and/or research question clearly reported/defined?	35 (44)	16 (20)	28 (35)	
Was there a clear statement of the aims and/or objectives of the research?	69 (87)	7 (9)	3 (4)	
Was a qualitative methodology appropriate?	63 (80)	16 (20)		
Was the research design appropriate to address the aims of the research?	66 (84)	8 (10)	2 (3)	3 (4)
Was the sampling and recruitment strategy clearly defined and justified?	44 (56)	24 (30)	9 (11)	2 (3)
Was the method of data collection well described?	57 (72)	18 (23)	4 (5)	
Were any techniques to enhance trustworthiness used?	38 (48)	12 (15)	15 (19)	14 (18)
Has the relationship between researchers and participants been adequately considered?	10 (13)	16 (20)	51 (65)	2 (3)
Have ethical issues been taken into consideration?	24 (30)	39 (49)	6 (8)	10 (13)
Was the data analysis/interpretation process well described and justified?	43 (54)	22 (28)	14 (18)	
Was there a clear statement of findings?	69 (87)	10 (13)		
Are the analysis and findings credible?	55 (70)	22 (28)	1 (1)	1 (1)
Was any conflict of interest reported?	4 (5)		75 (95)	

Four overarching themes and 10 subthemes were generated from our analysis. Our set of analytical themes did not align with a sequential pattern or predefined stages of implementation/adoption. Included papers rarely stated how ‘implementation’ was understood by researchers or the implementing organisation and, where present, definitions were extremely heterogeneous, ranging from examinations of the process in terms of specific time frames (eg, ref 41) through to its conceptualisation as an ongoing process (eg, refs 42 43). Hence, data were pooled on the basis of patterns of analytical concerns incorporating a wide range of conceptualisations about the implementation process. Table 2 summarises our coding framework, and online supplementary table 5 details the distribution of our themes by included papers. Themes are described below and exemplar data extracts provided in online supplementary table 6.

10.1136/bmjqs-2018-009082.supp6 Supplementary data

10.1136/bmjqs-2018-009082.supp7 Supplementary data

**Table 2:** Set of themes and subthemes generated from included papers

Themes and subthemes	Summary description
Contextualising the implementation and impact of electronic prescribing (ePrescribing) or computerised provider/physician order entry (CPOE) systems in hospitals	Authors’ descriptions/interpretations and primary data reporting on contextual factors and/or actions that that had taken place prior to system implementation.
Preparing the organisation for change	
Preparing stakeholders for change	
Factors affecting the implementation process of ePrescribing/CPOE systems	Authors’ descriptions/interpretations and primary data reporting on process-related issues.
Factors positively impacting the implementation process	
Factors negatively impacting the implementation process	
Positive and negative implications of ePrescribing/CPOE systems	Authors’ descriptions/interpretations and primary data reporting on impact-related issues in terms of benefits/problems, both in practice and/or at the organisational level.
Positive practice implications	
Negative practice implications	
Positive organisational implications	
Negative organisational implications	
Mixed impacts and change processes	Authors’ descriptions/interpretations and primary data reporting on impact-related issues that are not clear benefits/problems and/or focus on documenting change processes, both in practice and/or at the organisational levels.
Change in practice	
Change at the organisational level	

### Contextualising the implementation and impact of ePrescribing/CPOE in hospitals

Factors and/or actions undertaken prior to implementation were highlighted in some studies as important to understand the implementation and impact of ePrescribing/CPOE in hospitals. These illustrate the importance of allocating resources, prior to implementation, to prepare for both the organisational change and its stakeholders for changes in practice competencies and behaviour.

#### Preparing the organisation for change

A range of organisational factors were highlighted as a key enablers of successful implementation, including: defining an implementation strategy^44^; planning the change in terms of timescale, deliverability and organisational/structural needs (eg, IT networks and underlying drug database)^44–47^; understanding current practice and workflows and their variability^46 48 49^; building a good relationship between hospitals and system suppliers^44 46^; and being able to design a system to fit the workflow.^24^

#### Preparing stakeholders for change

The active involvement of stakeholder groups across the hospital setting was seen as important in ensuring successful implementation. This included accommodating the agendas of multiple stakeholder groups^44 46 47^ and establishing ad hoc multidisciplinary networks^47^ to develop pathways^48^ and appraise service requirements against systems options^45^ while ensuring that stakeholders’ needs were met.^43 44 50^

Broader contextual factors such as key policy changes that can trigger or support project initiation^47^ were also reported as relevant to facilitating and understanding successful implementation of ePrescribing/CPOE systems in hospital settings.

### Factors affecting the implementation process of ePrescribing/CPOE systems

#### Factors positively impacting the implementation process

Top-level leadership and support were seen as key enablers, particularly if they could: bring in an understanding of the wider context and outside pressures within which the organisation was operating^43 51–54^; establish effective governance strategies to support implementation across the organisation through committees and working groups^19 24 47 51 52^; and identify and address any anticipated/emerging problems and needs, such as those relating to guidance/pathway/policy development, estimation/identification of resources, setting realistic time frames, workflow/practice changes and training needs.^18 19 24 44 49 50 55–57^

The availability of leadership roles and/or championing individuals on the ground was also seen as a key enabler^42 58^ particularly as a way to facilitate longer term success by bringing about engagement^16 19 44 48 51–53 59^ and support^19 46 51 52 56^ across stakeholder groups from early implementation. Other engagement/support strategies during implementation included the provision of ongoing training opportunities^18 19 24 43 53 56^ which, in some cases, were seen to promote a sense of pride in mastering and helping to implement the system across stakeholder groups.^48^

Piloting and testing the system prior to full implementation was identified as important to ensure safety^44 46 55^ despite the risks associated with running two systems simultaneously (paper and electronic), typically minimising the transition from pilot to full implementation.^44 46^

#### Factors negatively impacting the implementation process

Problems were identified that could emerge *during* the implementation of ePrescribing/CPOE and hinder or negatively impact the implementation process. The nature of the reported issues was similar across studies, including core technical challenges (eg, appropriate infrastructure and availability of devices, issues relating to the usability of the system, alignment between system functionalities and hospital processes and interoperability issues with other systems in use)^41 43 55–57 60–64^ as well as personal challenges experienced by stakeholders (eg, insufficient training and support during implementation, fear of change and anxiety associated with expectations, unfamiliarity and inexperience with the newly implemented system and contradictions/conflicts resulting from recently changed roles, policies or pathways).^19 41 42 48 56 60 62 63^ These were seen as important because they could change attitudes towards the system during implementation^16 41 55^ and result in significant implementation delays^21 55^ or even deimplementation.^60^

### Positive and negative implications of ePrescribing/CPOE systems

#### Positive practice implications

Users’ experiences suggested a positive impact on safety, including a perceived reduction of medication-related incidents and adverse events after implementation,^16 65 66^ mainly due to improved accessibility^9 46 67^ and legibility^9 19 46 61 68–71^ of prescriptions. These benefits were echoed by patients.^72^ Easy, ‘on-the-spot’ access to detailed and comprehensive patient history information^18 52 61 73–75^ was also seen as an important benefit, which also improved continuity of care. For implementations involving or consisting of new clinical decision support systems, safety benefits were also linked to the ability to access built-in order sets and information on drugs and doses^9 67 73–77^ and the system’s ability to prevent dosing errors^9 45 47^ through an automatic alerting functionality^61 78–80^ at the time of prescribing.

Other reported benefits of eprescribing/CPOE related to perceived time-saving across the medication process: from faster prescribing and ordering of medications^9 18 24 69 75 81^ through to faster checking and supply of medicines.^46 66 69 70^ These time-saving benefits were afforded by a range of aspects brought in by ePrescribing/CPOE, such as the ability to access prescriptions remotely^9 18 24 47 48 51 52 61 69 73 74 81^ or improved legibility and completeness of prescriptions.^24 46 70^

Several studies also reported on a range of performance benefits other than strictly time-related efficiencies, including improvement in: coordination and communication,^18 42 47 52 70^ prescribing accuracy and timeliness^67 82^ and ability to easily find, prioritise and track orders.^9 67 70^

#### Negative practice implications

Most reported negative practice implications involved a range of perceived inefficiencies (eg, excessive complexity of screens to complete prescriptions and having to log in and out of multiple systems) with many increasing task-time across all stages of the medication process^9 16 18 51 52 61 67 72 73 79 81–84^ and/or increased workloads.^9 46 47 49 55 66 69 70 84–86^ In some cases system-related inefficiencies were still experienced 1 year after implementation,^18^ with some perceiving themselves to be back to baseline levels of efficiency at around 2 years postimplementation^81^ and others considered unlikely to ever return to pre-CPOE efficiency levels.^61^

The lack of appropriate IT infrastructure to ensure the smooth and responsive functioning of a system (eg, integration of coexisting systems, log-in and screen-loading times, availability of devices to interact with the system and provision of ongoing technical support to users) was seen to have disruptive consequences on health professionals’ workflows after the implementation of ePrescribing/CPOE.^9 24 47 48 51 52 61 68 75 80 84 86–88^

Negative practice implications were often perceived to counterbalance the benefits of ePrescribing/CPOE from a clinical perspective, particularly where the implementation of ePrescribing/CPOE was also associated with the introduction of new, unintended and often unanticipated safety risks.^9 14 18 19 42 43 49 61 63 65 68 71 73 75 76 78 81 83–86 88–93^ For example, a number of issues relating to systems’ interfaces and functionalities (such as excessive triggering of alerts, long lists of medication, default dosing functionality, limited dosing scales or forced sequences of field completion and navigation across screens, views and overviews) were perceived to increase the risk of specific errors.

#### Positive organisational implications

Although most included studies focused on benefits/problems of ePrescribing/CPOE in practice, some highlighted broader organisational issues.

Positive organisational implications had to do with the cost-effectiveness of the monitoring potential afforded by ePrecribing/CPOE technologies for quality and safety assurance purposes^45 48 51 52 77 93^ alongside its potential for financial efficiency^43 55 93^ and the positive impact on institutional reputation associated with being seen as a technologically advanced organisation.^43^

#### Negative organisational implications

Some studies reported a sense of distrust from clinicians towards some drivers expressed from a managerial perspective. For example, some studies reported clinicians perceiving ePrescribing/CPOE to be more advantageous to managers/administrators and imposed on them rather than driven by genuine clinical needs.^51 52^ Other studies reported clinicians’ concerns relating to the use of data generated by the ePrescribing/CPOE system for surveillance and performance management purposes.^20 77 93^

The lack of integration with other existing health information technology systems was perceived as a barrier to effective and reliable information transfer across coexisting systems in hospitals. Moreover, such lack of integration was seen to introduce risks (such as the potential for duplication associated with manual data entry across systems) that can hinder the availability of timely and complete data and compromise the ability of an organisation to realise the full potential of ePrescribing/CPOE systems.^23 43 49 51 55^

Other perceived problems included a lack of organisational policies, management practices and standards of practice that address/support new or changing procedures and workflows after implementation.^66 67 71 86^

### Mixed impacts and change processes

Beyond the benefits-and-problems rationale employed by most studies to describe the impact of implementing ePrescribing/CPOE in hospitals, papers also reported on what we have called ‘mixed impacts and change processes’, that is, impacts that cannot be easily framed as ‘positive’ or ‘negative’ per se but are better understood as ‘differences’ from whatever there was prior to implementation and as such they have the potential to result in either positive or negative implications, or both (or neither), in different contexts.

#### Change in practice

The main transformations reported by studies involved changes in work practices, particularly around workflows, interactions and communication:

- *Workflow-related transformations* included changes in aspects such as work pace, sequence and dynamics^18 57 81 85 88 93 94^ that can reshape the factors leading to medication errors^71 95^ and impact on many other specific aspects of everyday practice for doctors (eg, changes in the sequence and nature of cognitive tasks physicians undertake when admitting a patient to hospital)^96^ as well as nurses (eg, ability to document that a medication was given becomes subject to system access and log-in)^67 71^ and pharmacists (eg, shift in documentation and annotation practices, particularly due to systems’ built-in drug information).^70 71 97 98^
- *Interaction-related transformations* included a perceived increased interdependence resulting from changes in the frequency, volume and/or nature of staff-staff interactions (eg, between doctors and nurses or between pharmacists and doctors)^42 43 47 63 71 83 86 88 98 99^ and staff–patient interactions (eg, pharmacist–patient or doctor–patient interactions).^16 86 93 98 100^
- *Communication-related transformations* included changes in interprofessional communication patterns, task coordination and flow of information (eg, pharmacy–clinician or doctor–nurse communication, communication between administration and clinical staff and communication between shifts)^24 43 51 52 65 67 71 74 80 94 101–104^ including changes in the educational experiences in teaching/academic hospitals^105^ as well as changes in patient communication.^86 88 98 100 101^

These changes, alongside the need to accommodate idiosyncrasies of the systems themselves, were perceived to have shifted professional roles^47 63 65 71 79 83 88 95 97 98 102 103 106 107^ that often translated into the emergence of a wide range of workarounds^9 16 18 22 48 49 51 61 66 74 78 84 88 92 93 108^ across all the stages of the medication process.

#### Change at the organisational level

CPOE/ePrescribing systems were perceived to shift governance practices, bringing in new ways to handle and enact organisational power and organisational politics.^43 47 51 52 57 93^ For example, choosing a system and devising an implementation strategy can enable those leading on its implementation to influence the distribution of its advantages and disadvantages within the organisation^43^ by focusing more on particular processes’ or stakeholders’ needs over others (eg, doctors over pharmacists or managers over clinicians).^47 51 52 71^

Other studies reported how ePrescribing/CPOE systems enable the generation of, and access to, new data and metrics about individuals, teams, services and organisations^9 20 47 62 93^ to inform service evaluation and improvement, but with the proviso that appropriate strategies and resources for data monitoring, analysis and follow-up had to be in place to enable improvements.^62 92 109^

CPOE/ePrescribing systems can introduce or highlight discrepancies between established processes/policy/guidelines and practice under the newly implemented system^48 64 83 85 92 95^ the extent and nature of which are perceived and experienced differently across different clinical contexts.^51 52 94^ Such gaps were addressed by organisations by either performing modiﬁcations to the system to realign practice and processes/policy/guidelines^47 66 93^ and/or by making adjustments to current processes/policy/guidelines,^9 46 66 81^ including the temporary formalisation of emerging workarounds to mitigate known system limitations that were perceived as patient safety risks.^22^

## Discussion

We carried out a thematic synthesis of 79 papers to examine how stakeholders experience the adoption of ePrescribing/CPOE systems in hospitals.

Stakeholders’ perspectives revealed a mixed set of impacts that collectively do not clearly frame ePrescribing/CPOE as resulting in either an improvement or a deterioration of the quality and safety of hospital services. Instead, our findings reveal coexisting benefits and problems, which often overlap and counterbalance each other in the context of competing impacts and further-reaching, more complex changes. Taken together, these can be understood as an illustration of cultural shifts that reframe and recast the issues and challenges of the medication-related aspects of quality and safety in hospitals.^110^ Implementation strategies should explicitly and integrally address the change processes triggered by the adoption of ePrescribing/CPOE, both in practice and at the organisational levels, rather than focusing solely on discrete benefits/problems, recognising such changes are multifaceted, highly contingent on multiple perspectives and context dependent.

To address this, implementing organisations and teams could call on available implementation theories, models and frameworks^28 29^ to inform their implementation strategies, as well as research specifically addressing change processes and contextual factors involved in the adoption of ePrescribing/CPOE in hospitals. Although studies included in this review have largely focused on benefits/problems of ePrescribing/CPOE rather than the change processes underpinning them, these processes are well documented across included studies (eg, ref 22) and have the potential to inform future implementation strategies through a more comprehensive understanding of the impact of ePrescribing/CPOE.^5 71^

Only a few studies examined the implementation and impact of ePrescribing/CPOE taking into account factors and/or actions undertaken prior to implementation. However, these would suggest that more attention (and appropriate allocation of resources) to preimplementation considerations,^111 112^ including appropriate contextualisation of implementation strategies with specific reference to organisational and stakeholder groups needs and agendas, should facilitate successful implementation.^44 45^ Furthermore, echoing the sociotechnical nature of ePrescribing/CPOE, our findings suggest that assessing and responding to organisational and stakeholders’ needs should be treated as an ongoing, emergent feature of ePrescribing/CPOE adoption.

Our findings suggest drivers for implementing ePrescribing/CPOE in hospitals cannot be straightforwardly explained by the benefits experienced by those involved in their everyday use. Risks and safety concerns have been reported throughout the period covered by this review, in keeping with previous findings.^10^ While most included studies focused on clinicians’ perspectives, their needs have not been centrally addressed in ePrescribing/CPOE implementations. Conversely, little attention has been paid to the broader organisational issues, including potentially powerful drivers and factors from a managerial or health-systems perspective. Instead, most reported impacts were practice related. An in-depth knowledge of incentives and drivers of a political, financial, corporate or managerial nature could have helped explain why clinicians’ needs may not have been central to ePrescribing/CPOE implementations and contextualise the practice-related impacts of ePrescribing/CPOE adoption in hospitals, so that they can be better understood, explained and researched. It follows that organisational transparency on the intended direction of change in clinical practice and at the organisational level, and seeking, management and balancing of different stakeholder perspectives throughout ePrescribing/CPOE adoption journey, should help implementing organisations to address potential negative implications and promote beneficial contextual factors.

A further research gap was the limited number of studies drawing on patients’ or carers’ views.^21 45 62 72 89 100 109^ These could provide valuable insights related to key aspects of ePrescribing/CPOE systems in practice, such as shifts in communication/interaction patterns^100^ and the involvement of patients/carers in medication safety,^112^ including the examination of ePrescribing/CPOE as a potential barrier to patients/carers accessing their own prescriptions while in hospital. This is needed to understand the impact of ePrescribing/CPOE on enacting patient-centred care, in particular, the fundamental tenet of acknowledging and valuing patients’/carers’ experiential knowledge. Patients, particularly those with multimorbidity and polypharmacy, see their care managed under multiple or changing systems over time and/or across settings. Implementing organisations and teams should seek and address patient and carer views and experiences to ensure patient-centred care is maintained and patient satisfaction sustained during system implementation and optimisation.

Our review has limitations. Variable reporting quality of included papers reduced our ability to consider contextual information about specific settings and/or systems and to accurately assess quality. We did not carry out quantitative inter-reviewer reliability assessments. Instead, we ensured reliability and consistency across reviewers by systematically discussing all disagreements, involving additional reviewers when required to achieve consensus. In this secondary analysis, another important limitation was the restricted access to primary data: our findings draw on authors’ interpretations in articles’ results sections and any illustrative quotes reported to support them. We sought to provide an integrative understanding of ePrescribing/CPOE systems from stakeholders’ experiences drawing on multiple perspectives that have engaged from different angles with similar interventions in secondary care contexts. We noted if and how any key differences in characteristics (such as stakeholder or system type) translated into any salient aspects of this multiperspective narrative but acknowledge that a reporting focusing on these differences might also be of interest.

## Conclusions

The adoption of ePrescribing/CPOE in hospitals can be understood as cultural shifts that reframe the medication-related aspects of quality and safety, featuring coexisting benefits and problems. Implementing organisations and teams should consider the breadth and depth of changes that ePrescribing/CPOE adoption can trigger rather than focus on discrete benefits/problems and favour implementation strategies that: consider the preimplementation context; are responsive to (and transparent about) organisational and stakeholder needs and agendas; and can be sustained effectively over time as implementations develop and gradually transition to routine use and system optimisation. Alongside this, patients’ views and experiences should be sought throughout to ensure sustained patient satisfaction during system implementation and avoid unintended negative consequences on the organisations’ ability to enact patient-centred care.

## References

[1] Institute of Medicine Preventing medication errors. Washington, DC: The National Academies Press, 2007.

[2] Dean B, Schachter M, Vincent C, et al Causes of prescribing errors in hospital inpatients: a prospective study. The Lancet 2002;359:1373–8. 10.1016/S0140-6736(02)08350-2 11978334

[3] Tully MP, Ashcroft DM, Dornan T, et al The causes of and factors associated with prescribing errors in hospital inpatients. Drug Safety 2009;32:819–36. 10.2165/11316560-000000000-00000 19722726

[4] Keers RN, Williams SD, Cooke J, et al Causes of medication administration errors in hospitals: a systematic review of quantitative and qualitative evidence. Drug Saf 2013;36:1045–67. 10.1007/s40264-013-0090-2 23975331 PMC3824584

[5] Parry AM, Barriball KL, While AE Factors contributing to registered nurse medication administration error: a narrative review. Int J Nurs Stud 2015;52:403–20. 10.1016/j.ijnurstu.2014.07.003 25443300

[6] Bates DW, Gawande AA Improving safety with information technology. N Engl J Med 2003;348:2526–34. 10.1056/NEJMsa020847 12815139

[7] Black AD, Car J, Pagliari C, et al The impact of eHealth on the quality and safety of health care: a systematic overview. PLoS Med 2011;8:e1000387 10.1371/journal.pmed.1000387 21267058 PMC3022523

[8] Radley DC, Wasserman MR, Olsho LE, et al Reduction in medication errors in hospitals due to adoption of computerized provider order entry systems. J Am Med Inform Assoc 2013;20:470–6. 10.1136/amiajnl-2012-001241 23425440 PMC3628057

[9] Cresswell KM, Bates DW, Williams R, et al Evaluation of medium-term consequences of implementing commercial computerized physician order entry and clinical decision support prescribing systems in two 'early adopter' hospitals. J Am Med Inform Assoc 2014;21:e194–202. 10.1136/amiajnl-2013-002252 24431334 PMC4173168

[10] Ranji SR, Rennke S, Wachter RM Computerised provider order entry combined with clinical decision support systems to improve medication safety: a narrative review. BMJ Qual Saf 2014;23:773–80. 10.1136/bmjqs-2013-002165 24728888

[11] Barber N Electronic prescribing--safer, faster, better? J Health Serv Res Policy 2010;15(1_suppl):64–7. 10.1258/jhsrp.2009.09s109 20075133

[12] Redwood S, Rajakumar A, Hodson J, et al Does the implementation of an electronic prescribing system create unintended medication errors? A study of the sociotechnical context through the analysis of reported medication incidents. BMC Med Inform Decis Mak 2011;11:29 10.1186/1472-6947-11-29 21569397 PMC3116457

[13] Schiff GD, Hickman T-TT, Volk LA, et al Computerised prescribing for safer medication ordering: still a work in progress. BMJ Qual Saf 2016;25:315–9. 10.1136/bmjqs-2015-004677 26515444

[14] Mozaffar H, Cresswell KM, Williams R, et al Exploring the roots of unintended safety threats associated with the introduction of hospital ePrescribing systems and candidate avoidance and/or mitigation strategies: a qualitative study. BMJ Qual Saf 2017;26:722–33. 10.1136/bmjqs-2016-005879 28174319

[15] Niazkhani Z, Pirnejad H, Berg M, et al The impact of computerized provider order entry systems on inpatient clinical workflow: a literature review. J Am Med Inform Assoc 2009;16:539–49. 10.1197/jamia.M2419 19390113 PMC2705258

[16] Barber N, Cornford T, Klecun E Qualitative evaluation of an electronic prescribing and administration system. Quality and Safety in Health Care 2007;16:271–8. 10.1136/qshc.2006.019505 17693675 PMC2464937

[17] Sheikh A, Cornford T, Barber N, et al Implementation and adoption of nationwide electronic health records in secondary care in England: final qualitative results from prospective national evaluation in "early adopter" hospitals. BMJ 2011;343:d6054 10.1136/bmj.d6054 22006942 PMC3195310

[18] Abramson EL, Patel V, Malhotra S, et al Physician experiences transitioning between an older versus newer electronic health record for electronic prescribing. Int J Med Inform 2012;81:539–48. 10.1016/j.ijmedinf.2012.02.010 22465355

[19] Simon SR, Keohane CA, Amato M, et al Lessons learned from implementation of computerized provider order entry in 5 community hospitals: a qualitative study. BMC Med Inform Decis Mak 2013;13:67 10.1186/1472-6947-13-67 23800211 PMC3695777

[20] Dixon-Woods M, Redwood S, Leslie M, et al Improving quality and safety of care using "technovigilance": an ethnographic case study of secondary use of data from an electronic prescribing and decision support system. Milbank Q 2013;91:424–54. 10.1111/1468-0009.12021 24028694 PMC3790520

[21] Mozaffar H, Cresswell KM, Lee L, et al Taxonomy of delays in the implementation of hospital computerized physician order entry and clinical decision support systems for prescribing: a longitudinal qualitative study. BMC Med Inform Decis Mak 2016;16:25 10.1186/s12911-016-0263-x 26911288 PMC4766744

[22] Cresswell KM, Mozaffar H, Lee L, et al Workarounds to hospital electronic prescribing systems: a qualitative study in English hospitals. BMJ Qual Saf 2017;26:542–51. 10.1136/bmjqs-2015-005149 27129493

[23] Cresswell KM, Mozaffar H, Lee L, et al Safety risks associated with the lack of integration and Interfacing of hospital health information technologies: a qualitative study of hospital electronic prescribing systems in England. BMJ Qual Saf 2017;26:530–41. 10.1136/bmjqs-2015-004925 27037303

[24] Ash JS, Gorman PN, Hersh WR, et al Perceptions of house officers who use physician order entry. Proc AMIA Symp 1999:471–5. 10566403 PMC2232743

[25] Farre A, Bem D, Heath G, et al Perceptions and experiences of the implementation, management, use and optimisation of electronic prescribing systems in hospital settings: protocol for a systematic review of qualitative studies. BMJ Open 2016;6:e011858 10.1136/bmjopen-2016-011858 PMC4947719 27401366

[26] Hogan-Murphy D, Tonna A, Strath A, et al Healthcare professionals’ perceptions of the facilitators and barriers to implementing electronic systems for the prescribing, dispensing and administration of medicines in hospitals: a systematic review. Eur J Hosp Pharm 2015;22:358–65. 10.1136/ejhpharm-2015-000722

[27] Linton JD Implementation research: state of the art and future directions. Technovation 2002;22:65–79. 10.1016/S0166-4972(01)00075-X

[28] Damschroder LJ, Aron DC, Keith RE, et al Fostering implementation of health services research findings into practice: a consolidated framework for advancing implementation science. Implement Sci 2009;4 10.1186/1748-5908-4-50 PMC2736161 19664226

[29] May C, Finch T Implementing, embedding, and integrating practices: an outline of normalization process theory. Sociology 2009;43:535–54. 10.1177/0038038509103208

[30] Tong A, Flemming K, McInnes E, et al Enhancing transparency in reporting the synthesis of qualitative research: ENTREQ. BMC Med Res Methodol 2012;12:181 10.1186/1471-2288-12-181 23185978 PMC3552766

[31] Thomas J, Harden A Methods for the thematic synthesis of qualitative research in systematic reviews. BMC Med Res Methodol 2008;8:45 10.1186/1471-2288-8-45 18616818 PMC2478656

[32] University of Texas School of Public Health Search filters for qualitative studies. Available: http://libguides.sph.uth.tmc.edu/ovid_medline_filters [Accessed 18 Feb 2016].

[33] SBU Evaluation and synthesis of studies using qualitative methods of analysis. Stockholm: Swedish Agency for Health Technology Assessment and Assessment of Social Services (SBU), 2014.

[34] Cresswell K, Mozaffar H, Shah S, et al Approaches to promoting the appropriate use of antibiotics through Hospital electronic prescribing systems: a scoping review. Int J Pharm Pract 2017;25:5–17. 10.1111/ijpp.12274 27198585

[35] Gagnon M-P, Nsangou Édith-Romy, Payne-Gagnon J, et al Barriers and facilitators to implementing electronic prescription: a systematic review of user groups' perceptions. J Am Med Inform Assoc 2014;21:535–41. 10.1136/amiajnl-2013-002203 24130232 PMC3994867

[36] Boonstra A, Versluis A, Vos JFJ Implementing electronic health records in hospitals: a systematic literature review. BMC Health Serv Res 2014;14:370 10.1186/1472-6963-14-370 25190184 PMC4162964

[37] Critical Appraisal Skills Programme (CASP) Qualitative research checklist, 2013 Available: http://www.casp-uk.net/ [Accessed 11 Dec 2015].

[38] O'Brien BC, Harris IB, Beckman TJ, et al Standards for reporting qualitative research: a synthesis of recommendations. Acad Med 2014;89:1245–51. 10.1097/ACM.0000000000000388 24979285

[39] Dixon-Woods M et al The problem of appraising qualitative research. Quality and Safety in Health Care 2004;13:223–5. 10.1136/qshc.2003.008714 15175495 PMC1743851

[40] Carroll C, Booth A, Lloyd-Jones M Should we exclude inadequately reported studies from qualitative systematic reviews? an evaluation of sensitivity analyses in two case study reviews. Qual Health Res 2012;22:1425–34. 10.1177/1049732312452937 22865107

[41] Malato LA, Kim S End-User perceptions of a computerized medication system: is there resistance to change? Journal of health and human services administration 2004;27:34–55. 15962576

[42] Ash JS, Gorman PN, Lavelle M, et al Perceptions of physician order entry: results of a cross-site qualitative study. Methods of information in medicine 2003;42:313–23. 14534628

[43] Ash JS, Gorman PN, Lavelle M, et al A cross-site qualitative study of physician order entry. Journal of the American Medical Informatics Association 2003;10:188–200. 10.1197/jamia.M770 12595408 PMC150372

[44] Cresswell K, Coleman J, Slee A, et al Investigating and learning lessons from early experiences of implementing ePrescribing systems into NHS hospitals: a questionnaire study. PLoS One 2013;8:e53369 10.1371/journal.pone.0053369 23335961 PMC3546047

[45] Cresswell KM, Slee A, Coleman J, et al Qualitative analysis of round-table discussions on the business case and procurement challenges for hospital electronic prescribing systems. PLoS One 2013;8:e79394 10.1371/journal.pone.0079394 24260213 PMC3834189

[46] Cornford T, Savage I, Jani Y, et al Learning lessons from electronic prescribing implementations in secondary care 2010:233–7. 20841684

[47] Davidson EJ, Chismar WG The interaction of institutionally triggered and technology-triggered social structure change: an investigation of computerized physician order entry. MIS Quarterly 2007;31:739–58. 10.2307/25148818

[48] Ash JS, Gorman PN, Lavelle M, et al Multiple perspectives on physician order entry. Proceedings / AMIA. Annual Symposium AMIA Symposium 2000:27–31. PMC2243815 11079838

[49] Jeon J, Taneva S, Kukreti V, et al Toward successful migration to computerized physician order entry for chemotherapy. Current Oncology 2014;21:221–8. 10.3747/co.21.1759 PMC3997455 24764707

[50] Pirnejad H, Niazkhani Z, Aarts J, et al What makes an information system more preferable for clinicians? A qualitative comparison of two systems. Stud Health Technol Inform 2011;169:392–6. 21893779

[51] Ash J, Gorman P, Lavelle M, et al Investigating physician order entry in the field: lessons learned in a multi- study. Stud Health Technol Inform 2001;84:1107–11. 11604900

[52] Ash JS, Sittig DF, Seshadri V, et al Adding insight: a qualitative cross-site study of physician order entry. Int J Med Inform 2005;74:623–8. 10.1016/j.ijmedinf.2005.05.005 15964780 PMC1524826

[53] Ash JS, McCormack JL, Sittig DF, et al Standard practices for computerized clinical decision support in community hospitals: a national survey. J Am Med Inform Assoc 2012;19:980–7. 10.1136/amiajnl-2011-000705 22707744 PMC3486730

[54] Botta MD, Cutler DM Meaningful use: floor or ceiling? Health Care 2014;2:48–52. 10.1016/j.hjdsi.2013.12.011 26250089

[55] Aarts J, Doorewaard H, Berg M Understanding implementation: the case of a computerized physician order entry system in a large Dutch University medical center. J Am Med Inform Assoc 2004;11:207–16. 10.1197/jamia.M1372 14764612 PMC400519

[56] Hardie R-A, Baysari MT, Lake R, et al User perceptions of the implementation of an electronic medication management system in a paediatric setting. Stud Health Technol Inform 2017;239:41–7. 28756435

[57] Ash JS, Lyman J, Carpenter J, et al A diffusion of innovations model of physician order entry. Proc AMIA Symp 2001:22–6. 11825150 PMC2243456

[58] Ash J et al Implementing computerized physician order entry: the importance of special people. Int J Med Inform 2003;69:235–50. 10.1016/S1386-5056(02)00107-7 12810127

[59] Cresswell KM, Lee L, Mozaffar H, et al Sustained user engagement in health information technology: the long road from implementation to system optimization of computerized physician order entry and clinical decision support systems for prescribing in hospitals in England. Health Serv Res 2017;52:1928–57. 10.1111/1475-6773.12581 27714800 PMC5583302

[60] Griffon N, Schuers M, Joulakian M, et al Physician satisfaction with transition from CPOE to paper-based prescription. Int J Med Inform 2017;103:42–8. 10.1016/j.ijmedinf.2017.04.007 28551000

[61] Baysari MT, Hardie R-A, Lake R, et al Longitudinal study of user experiences of a CPOE system in a pediatric hospital. Int J Med Inform 2018;109:5–14. 10.1016/j.ijmedinf.2017.10.018 29195706

[62] Cresswell K, Smith P, Swainson C, et al Establishing data-intensive healthcare: the case of hospital electronic prescribing and medicines administration systems in Scotland. J Innov Health Inform 2016;23:572–9. 10.14236/jhi.v23i3.842 28059691

[63] Puaar SJ, Franklin BD Impact of an inpatient electronic prescribing system on prescribing error causation: a qualitative evaluation in an English Hospital. BMJ Qual Saf 2018;27:529–38. 10.1136/bmjqs-2017-006631 29018058

[64] Mozaffar H, Williams R, Cresswell K, et al The evolution of the market for commercial computerized physician order entry and computerized decision support systems for prescribing. J Am Med Inform Assoc 2016;23:349–55. 10.1093/jamia/ocv095 26338217 PMC11740538

[65] Mills PR, Weidmann AE, Stewart D Hospital staff views of prescribing and discharge communication before and after electronic prescribing system implementation. Int J Clin Pharm 2017;39:1320–30. 10.1007/s11096-017-0543-2 29076013 PMC5694510

[66] Yang Z, Ng B-Y, Kankanhalli A, et al Workarounds in the use of is in healthcare: a case study of an electronic medication administration system. Int J Hum Comput Stud 2012;70:43–65. 10.1016/j.ijhcs.2011.08.002

[67] Tschannen D, Talsma A, Reinemeyer N, et al Nursing medication administration and workflow using computerized physician order entry. CIN: Computers, Informatics, Nursing 2011;29:401–10. 10.1097/NCN.0b013e318205e510 21164338

[68] Riccioli C, Cacciabue PC, Campanini M, et al Designing, implementing and evaluating e-prescription: a field study and comparison with psiP results. Stud Health Technol Inform 2011;166:105–15. 21685616

[69] Holden RJ Cognitive performance-altering effects of electronic medical records: an application of the human factors paradigm for patient safety. Cogn Tech Work 2011;13:11–29. 10.1007/s10111-010-0141-8 PMC3072581 21479125

[70] McMullen CK, Macey TA, Pope J, et al Effect of computerized prescriber order entry on pharmacy: experience of one health system. Am J Health Syst Pharm 2015;72:133–42. 10.2146/ajhp140106 25550137

[71] Hawkins SF, Nickman NA, Morse JM The paradox of safety in medication management. Qual Health Res 2017;27:1910–23. 10.1177/1049732317732968 28992756

[72] O’Grady K, Donyai P, Franklin BD Patients’ views about an electronic prescribing and drug administration system in secondary care. British Journal of Healthcare Computing & Information Management 2006;23:15–18.

[73] Baysari MT, Westbrook JI, Day RO Understanding doctors' perceptions of their prescribing competency and the value they ascribe to an electronic prescribing system. Stud Health Technol Inform 2012;178:1–6. 22797011

[74] Pirnejad H, Niazkhani Z, van der Sijs H, et al Evaluation of the impact of a CPOE system on nurse-physician communication--a mixed method study. Methods Inf Med 2009;48:350–60. 10.3414/ME0572 19448880

[75] Omar A, Ellenius J, Lindemalm S Evaluation of electronic prescribing decision support system at a tertiary care pediatric Hospital: the user acceptance perspective. Stud Health Technol Inform 2017;234:256–61. 28186051

[76] Santucci W, Day RO, Baysari MT Evaluation of hospital-wide computerised decision support in an intensive care unit: an observational study. Anaesth Intensive Care 2016;44:507–12. 10.1177/0310057X1604400403 27456183

[77] Redwood S, Ngwenya NB, Hodson J, et al Effects of a computerized feedback intervention on safety performance by junior doctors: results from a randomized mixed method study. BMC Med Inform Decis Mak 2013;13:63 10.1186/1472-6947-13-63 23734871 PMC3704711

[78] Baysari MT, Westbrook JI, Richardson K, et al Optimising computerised alerts within electronic medication management systems: a synthesis of four years of research 2014. 25087519

[79] Jung M, Hoerbst A, Hackl WO, et al Attitude of physicians towards automatic alerting in computerized physician order entry systems. A comparative international survey. Methods Inf Med 2013;52:99–108. 10.3414/ME12-02-0007 23187311

[80] Baysari MT, Westbrook JI, Richardson KL, et al The influence of computerized decision support on prescribing during ward-rounds: are the decision-makers targeted? J Am Med Inform Assoc 2011;18:754–9. 10.1136/amiajnl-2011-000135 21676939 PMC3197993

[81] Abramson EL, Patel V, Pfoh ER, et al How physician perspectives on E-Prescribing evolve over time. Appl Clin Inform 2016;7:994–1006. 27786335 10.4338/ACI-2016-04-RA-0069 PMC5228140

[82] Holden RJ Physicians’ beliefs about using EMR and CPOE: In pursuit of a contextualized understanding of health IT use behavior. Int J Med Inform 2010;79:71–80. 10.1016/j.ijmedinf.2009.12.003 20071219 PMC2821328

[83] Carpenter JD, Gorman PN What’s so special about medications: a pharmacist’s observations from the POE study. Proc AMIA Symp 2001:95–9. 11825161 PMC2243687

[84] Niazkhani Z, Pirnejad H, van der Sijs H, et al Evaluating the medication process in the context of CPOE use: the significance of working around the system. Int J Med Inform 2011;80:490–506. 10.1016/j.ijmedinf.2011.03.009 21555237

[85] Campbell EM, Guappone KP, Sittig DF, et al Computerized provider order entry adoption: implications for clinical workflow. J Gen Intern Med 2009;24:21–6. 10.1007/s11606-008-0857-9 19020942 PMC2607519

[86] Wentzer HS, Böttger U, Boye N Unintended transformations of clinical relations with a computerized physician order entry system. Int J Med Inform 2007;76:S456–61. 10.1016/j.ijmedinf.2007.07.007 17936681

[87] Niès J, Pelayo S From users involvement to users’ needs understanding: A case study. Int J Med Inform 2010;79:e76–82. 10.1016/j.ijmedinf.2009.06.007 19660983

[88] Debono D, Taylor N, Lipworth W, et al Applying the theoretical domains framework to identify barriers and targeted interventions to enhance nurses' use of electronic medication management systems in two Australian hospitals. Implement Sci 2017;12 10.1186/s13012-017-0572-1 PMC5368903 28347319

[89] Savage I, Cornford T, Klecun E, et al Medication errors with electronic prescribing (eP): two views of the same picture. BMC Health Serv Res 2010;10:135 10.1186/1472-6963-10-135 20497532 PMC2890639

[90] Campbell EM, Sittig DF, Guappone KP, et al Overdependence on technology: an unintended adverse consequence of computerized provider order entry. AMIA Annu Symp Proc 2007:94–8. 18693805 PMC2710605

[91] van der Sijs H, Aarts J, van Gelder T, et al Turning off frequently overridden drug alerts: limited opportunities for doing it safely. J Am Med Inform Assoc 2008;15:439–48. 10.1197/jamia.M2311 18436915 PMC2442265

[92] Baysari M, Gigante JD, Moran M, et al Redesign of computerized decision support to improve antimicrobial prescribing. A controlled before-and-after study. Appl Clin Inform 2017;08:949–63. 10.4338/ACI2017040069 PMC6220696 28905978

[93] Shemilt K, Morecroft CW, Ford JL, et al Inpatient prescribing systems used in NHS acute trusts across England: a managerial perspective. Eur J Hosp Pharm Sci Pract 2017;24:213–7. 10.1136/ejhpharm-2016-000905 31156943 PMC6451506

[94] Niazkhani Z et al CPOE in non-surgical versus surgical specialties: a qualitative comparison of clinical contexts in the medication process. Open Med Inform J 2010;4:206–13. 10.2174/1874431101004010206 21594008 PMC3096890

[95] Abraham J, Kannampallil TG, Jarman A, et al Reasons for computerised provider order entry (CPOE)-based inpatient medication ordering errors: an observational study of voided orders. BMJ Qual Saf 2018;27:299–307. 10.1136/bmjqs-2017-006606 28698381

[96] Johnson CD, Zeiger RF, Das AK, et al Task analysis of writing hospital admission orders: evidence of a problem-based approach. AMIA Annu Symp Proc 2006:389–93. 17238369 PMC1839659

[97] Burgin A, O'Rourke R, Tully MP Learning to work with electronic patient records and prescription charts: experiences and perceptions of hospital pharmacists. Research in Social and Administrative Pharmacy 2014;10:741–55. 10.1016/j.sapharm.2013.11.005 24378236

[98] Burgin A, O’Rourke R, Tully M Learning to work with computerisation of medical notes and prescription charts. International Journal of Pharmacy Practice 2012;20.

[99] Pelayo S, Anceaux F, Rogalski J, et al Does CPOE actually disrupt physicians-nurses communications? 2010:173–7. 20841672

[100] Garfield S, Jheeta S, Husson F, et al The role of hospital inpatients in supporting medication safety: a qualitative study. PLoS One 2016;11:e0153721 10.1371/journal.pone.0153721 27093438 PMC4836703

[101] Dykstra R Computerized physician order entry and communication: reciprocal impacts. Proc AMIA Symp 2002:230–4. 12463821 PMC2244295

[102] Niazkhani Z, Pirnejad H, de Bont A, et al Evaluating inter-professional work support by a computerized physician order entry (CPOE) system. Stud Health Technol Inform 2008;136:321–6. 18487751

[103] Niazkhani Z, Pirnejad H, van der Sijs H, et al Computerized provider order entry system--does it support the inter-professional medication process? Lessons from a Dutch academic hospital. Methods Inf Med 2010;49:20–7. 10.3414/ME0631 19448890

[104] Pirnejad H, Niazkhani Z, van der Sijs H, et al Impact of a computerized physician order entry system on nurse–physician collaboration in the medication process. Int J Med Inform 2008;77:735–44. 10.1016/j.ijmedinf.2008.04.001 18514020

[105] Wong B, Kuper A, Robinson N, et al Computerised provider order entry and residency education in an academic medical centre: computerised provider order entry and medical education. Medical Education 2012;46:795–806. 22803757 10.1111/j.1365-2923.2012.04317.x

[106] Chow A, Lye DCB, Arah OA Psychosocial determinants of physicians’ acceptance of recommendations by antibiotic computerised decision support systems: A mixed methods study. Int J Antimicrob Agents 2015;45:295–304. 10.1016/j.ijantimicag.2014.10.009 25434998

[107] Sittig DF, Krall M, Kaalaas-Sittig J, et al Emotional aspects of computer-based provider order entry: a qualitative study. J Am Med Inform Assoc 2005;12:561–7. 10.1197/jamia.M1711 15905478 PMC1205605

[108] Zhou X, Ackerman M, Zheng K CPOE workarounds, boundary objects, and assemblages. In: Proceedings of the SIGCHI Conference on Human Factors in Computing Systems. ACM 2011:3353–62.

[109] Cresswell K, Coleman J, Smith P, et al Qualitative analysis of multi-disciplinary round-table discussions on the acceleration of benefits and data analytics through Hospital electronic prescribing (ePrescribing) systems. J Innov Health Inform 2016;23:501–9. 10.14236/jhi.v23i2.178 27869580

[110] Mannion R, Davies H Understanding organisational culture for healthcare quality improvement. BMJ 2018;363 10.1136/bmj.k4907 PMC6260242 30487286

[111] Farre A, Heath G, Shaw K, et al The role of paediatric nurses in medication safety prior to the implementation of electronic prescribing: a qualitative case study. J Health Serv Res Policy 2017;22:99–106. 10.1177/1355819616686995 28429973

[112] Farre A, Shaw K, Heath G, et al On doing ‘risk work’ in the context of successful outcomes: exploring how medication safety is brought into action through health professionals’ everyday working practices. Health Risk Soc 2017;19:209–25. 10.1080/13698575.2017.1336512

