# Supplementary material for: How do stakeholders experience the adoption of electronic prescribing systems in hospitals? A systematic review and thematic synthesis of qualitative studies
Source: BMJ Qual Saf. 2019 Jul 29;28(12):1021–31. doi: 10.1136/bmjqs-2018-009082 (PMC6934241; doi:10.1136/bmjqs-2018-009082)
Supplement: Supplementary data [file bmjqs-2018-009082supp001.pdf]

Supplementary Appendix 1: Example search strategy for Ovid MEDLINE(R)

1 Electronic Prescribing/
2 ("electronic prescribing" or e-prescribing or eprescribing).ti,ab.
3 Medical Order Entry Systems/ or Medication Systems, Hospital/ or Pharmacy service, Hospital/
4 ((medica$ or electronic or prescri$) adj3 system$1).ti,ab.
5 (computeri?ed adj2 ("order entry" or "order management")).ti,ab.
6 (CPOE or EP or EPMA or HEPMA or CDS or CDSS or eRx).ti,ab.
7 clinical decision support.mp. or Decision Support Systems, Clinical/
8 1 or 2 or 3 or 4 or 5 or 6 or 7
9 (perception$1 or experience$1 or perspective$1 or insight$1 or view$1 or opinion$1 or thought$1 or expectation$1 or belie$ or anticipation$1 or attitude$1 or prospect$1 or presumption$1 or observation$1 or lesson$1 or interaction$1).ti,ab.
10 8 and 9
11 limit 10 to "qualitative (maximizes specificity)"
12 qualitative research.mp. or exp Qualitative Research/
13 focus group$1.mp. or exp Focus Groups/
14 nursing methodology research.mp. or exp Nursing Methodology Research/
15 (qualitative or "grounded theory" or ethnogra$ or ethnolog$ or phenomenogra$ or phenomenolog$ or hermeneutic$ or "focus group$1" or "field study" or fieldwork or "field work" or narrat$ or "lived experience$1" or "life experience$1" or "key informant$1" or "mixed method$" or "multi-method$").ti,ab.
16 (("semi-structured" or semistructured or unstructured or informal or "in-depth" or indepth or "face-to-face" or structured or guide$) adj3 (interview$1 or discussion$1 or questionnaire$1)).ti,ab.
17 12 or 13 or 14 or 15or 16
18 10 and 17
19 11 or 18
