# Supplementary material for: How do stakeholders experience the adoption of electronic prescribing systems in hospitals? A systematic review and thematic synthesis of qualitative studies
Source: BMJ Qual Saf. 2019 Jul 29;28(12):1021–31. doi: 10.1136/bmjqs-2018-009082 (PMC6934241; doi:10.1136/bmjqs-2018-009082)
Supplement: Supplementary data [file bmjqs-2018-009082supp002.pdf]

Supplementary Table 1. Quality appraisal form items

**Approach and design**					
1	**Was the research problem and/or research question clearly reported/defined?**<br>• *Description and significance of the problem/phenomenon studied?*<br>• *Is there an explicit formulation of a research question or a statement drawing on a SPICE/PICO structure?*	**Yes**	**Partially**	**No**	**Unclear**
2	**Was there a clear statement of the aims and/or objectives of the research?**<br>• *Did the researchers explicitly state the specific research objectives?*	**Yes**	**Partially**	**No**	**Unclear**
3	**Was a qualitative methodology appropriate?**<br>• *Did the study seek to interpret or illuminate the actions and/or subjective experiences of research participants?*	**Yes**	**Partially**	**No**	**Unclear**
4	**Was the research design appropriate to address the aims of the research?**<br>• *Did the researcher justify the research design (e.g. have they discussed how they decided which method to use)?*	**Yes**	**Partially**	**No**	**Unclear**
5	**Was the sampling and recruitment strategy clearly defined and justified?**<br>• *Did the researchers explain how and why the participants were selected?*<br>• *Is there information about participant characteristics and if/why any participants chose not to take part?*<br>• *Was saturation discussed/achieved?*	**Yes**	**Partially**	**No**	**Unclear**
**Data collection**					
6	**Was the method of data collection well described?**<br>• *Was the setting justified?*<br>• *Is it clear what methods used to collect data?*<br>• *Were methods justified and made explicit?*<br>• *Were methods modified during the study (how, why)?*	**Yes**	**Partially**	**No**	**Unclear**
7	**Were any techniques to enhance trustworthiness used?**<br>• *e.g. member checking, audit trail, triangulation*	**Yes**	**Partially**	**No**	**Unclear**
8	**Has the relationship between researchers and participants been adequately considered?**<br>• *Did the researchers critically examine their own role, bias and influence?*<br>• *Did the researchers consider the implications of any changes in the research design?*<br>• *Were there any potential power relationships involved?*	**Yes**	**Partially**	**No**	**Unclear**
9	**Have ethical issues been taken into consideration?**	**Yes**	**Partially**	**No**	**Unclear**

	• *How was the research explained to participants?*<br>• *Did the researchers discuss issues around informed consent or confidentiality or how they handled any effects of the study on participants during/after the study?*<br>• *Was ethical approval sought?*				
	**Data analysis and findings**				
**10**	**Was the data analysis/interpretation process well described and justified?**<br>• *Is there a detailed description of the analysis process?*<br>• *Is the process by which inferences/categories/themes were derived from the data clearly explained?*	**Yes**	**Partially**	**No**	**Unclear**
**11**	**Was there a clear statement of findings?**<br>• *Is there adequate and balanced discussion of evidence both for and against the researchers' arguments?*<br>• *Are the findings discussed in relation to the original research question and/or study objectives?*	**Yes**	**Partially**	**No**	**Unclear**
**12**	**Are the analysis and findings credible?**<br>• *Are sufficient data presented to support the findings?*<br>• *To what extent contradictory data are taken into account?*<br>• *Did the researchers discuss the credibility of their findings*<br>• *Are the explanations presented plausible and coherent?*<br>• *Were alternative explanations for the findings explored?*	**Yes**	**Partially**	**No**	**Unclear**
	**Other**				
**13**	**Was any conflict of interest reported?**	**Yes**		**No**	
**14**	**Open comments for discussion**<br>• *How valuable is the research overall?*<br>• *Did the researchers discuss the contribution the study makes to existing knowledge or understanding?*<br>• *Were the findings considered in relation to current practice or policy?*<br>• *Did the researchers identify any new areas where further research is necessary?*<br>• *Did the researchers discuss whether or how the findings might be transferred to other populations/settings?*				
