# Supplementary material for: How do stakeholders experience the adoption of electronic prescribing systems in hospitals? A systematic review and thematic synthesis of qualitative studies
Source: BMJ Qual Saf. 2019 Jul 29;28(12):1021–31. doi: 10.1136/bmjqs-2018-009082 (PMC6934241; doi:10.1136/bmjqs-2018-009082)
Supplement: Supplementary data [file bmjqs-2018-009082supp003.pdf]

Supplementary Table 2. Data extraction form items

1	Full article reference
2	Study country
3	Study year(s)
4	Study setting
5	System type
6	Type of participants/user groups
7	Number of participants
8	Sampling strategy
9	Data collection methods
10	Data analysis methods
11	Theoretical framework
12	Reflexivity
13	Techniques to enhance trustworthiness
14	Study findings ('results' or 'findings' section in full)
