# Supplementary material for: How do stakeholders experience the adoption of electronic prescribing systems in hospitals? A systematic review and thematic synthesis of qualitative studies
Source: BMJ Qual Saf. 2019 Jul 29;28(12):1021–31. doi: 10.1136/bmjqs-2018-009082 (PMC6934241; doi:10.1136/bmjqs-2018-009082)
Supplement: Supplementary data [file bmjqs-2018-009082supp004.pdf]

Supplementary Table 3: Key characteristics of the included studies

Paper	Country	Setting	System type	Participants/user groups	Number of participants	Data collection method	Theoretical framework	Data analysis method
Aarts et al. (2004) [55]	Netherlands	Large academic medical center	Computerised Provider/Physician Order Entry (CPOE)	Project leaders<br>Project coordinators<br>Clinicians<br>IT administrators<br>Pathology technicians<br>Medical record coordinator	10	Semistructured interviews<br>Observations<br>Document analysis	Drawing on three theoretical aspects of science and technology studies (STS): sociotechnical approach; emergent change; success & failure	Content analysis
Abraham et al. (2018) [95]	United States	One academic medical centre	CPOE + Clinical decision support (CDS)	Physicians<br>Pharmacists<br>Nurses	80	Semistructured interviews	Data analysis was informed by a structured framework of CPOE-related medication errors (Schiff et al., 2015)	Thematic analysis
Abramson et al. (2012) [18]	United States	Urban hospital-based adult internal medicine outpatient clinic	Electronic health records (EHR)-based Electronic prescribing (ePrescribing) + CDS	Physicians	16	Observations<br>Semi-structured interviews		Grounded theory
Abramson et al. (2016) [81]	United States	Urban hospital-based adult internal medicine outpatient clinic	EHR-based ePrescribing + CDS	Physicians	13	Semi-structured interviews		Grounded theory
Ash et al. (1999) [24]	United States	Two hospitals, with system in use for 10 years and 2 years respectively	CPOE	Physicians<br>Administrators<br>Information technology staff<br>Pharmacists<br>Nurses	Not reported	Participant observation<br>Focus groups<br>Oral history interviews<br>Informal interviews		Grounded theory

Paper	Country	Setting	System type	Participants/user groups	Number of participants	Data collection method	Theoretical framework	Data analysis method
Ash et al. (2000) [48]	United States	Four hospitals across three organisations, with differences in teaching status, geography and length of system use.	CPOE	Physicians<br>Administrators<br>Information technology staff<br>Pharmacists<br>Nurses	Not reported	Participant observation<br>Focus groups<br>Oral history interviews<br>Informal interviews		Grounded theory
Ash et al. (2001a) [51]	United States	Four hospitals across three organisations, with differences in teaching status, geography and length of system use.	CPOE	Physicians<br>Administrators<br>Information technology staff<br>Pharmacists<br>Nurses	Not reported	Participant observation<br>Focus groups<br>Oral history interviews<br>Informal interviews		Grounded theory
Ash et al. (2001b) [57]	United States	Four hospitals across three organisations, with differences in teaching status, geography and length of system use.	CPOE	Clinicians<br>IT professionals<br>Administrators	55	Participant observation<br>Focus groups<br>Oral history interviews<br>Informal interviews	Diffusion of innovations theory framework over the results of grounded theory analysis	Grounded theory
Ash et al. (2003a) [43]	United States	Four hospitals across three organisations, with differences in teaching status, geography and length of system use.	CPOE	Clinicians<br>IT professionals<br>Administrators	56	Participant observation<br>Focus groups<br>Oral history interviews<br>Informal interviews		Grounded theory

Paper	Country	Setting	System type	Participants/user groups	Number of participants	Data collection method	Theoretical framework	Data analysis method
Ash et al. (2003b) [42]	United States	Four hospitals across three organisations, with differences in teaching status, geography and length of system use.	CPOE	Clinicians<br>IT professionals<br>Administrators	87	Participant observation<br>Focus groups<br>Oral history interviews<br>Informal interviews		Grounded theory
Ash et al. (2003c) [58]	United States	Four hospitals across three organisations, with differences in teaching status, geography and length of system use.	CPOE	Clinicians<br>IT professionals<br>Administrators	156	Participant observation<br>Focus groups<br>Oral history interviews<br>Informal interviews		Grounded theory
Ash et al. (2005) [52]	United States	Five hospitals across four organisations, with differences in teaching status, geography and length of system use.	CPOE	Clinicians<br>IT professionals<br>Administrators	not reported	Participant observation<br>Focus groups<br>Oral history interviews<br>Informal interviews		Grounded theory
Ash et al. (2012) [53]	United States	Thirty-four community hospitals, with over 5 years experience with CPOE each	CPOE + CDS	Clinical application coordinators<br>Chief medical information officers<br>Chief information officers<br>Clinical analysts<br>Department directors	34	Semistructured interview, conducted as a subset of questions in the context of a mixed-methods interview instrument		Grounded theory

Paper	Country	Setting	System type	Participants/user groups	Number of participants	Data collection method	Theoretical framework	Data analysis method
Barber et al. (2007) [16]	United Kingdom	Surgical ward in a teaching hospital	ePrescribing + limited CDS	Nursing staffDoctorsPharmacistsHospital management	40	InterviewsObservationsFocus group	Socio-technical evaluation framework	Discourse analysis
Baysari et al. (2011) [80]	Australia	Teaching hospital	CPOE + CDS	Specialty teams (cardiology, clinical pharmacology, lung transplantation, colorectal surgery, gastroenterology, gerontology, hematology, infectious diseases, nephrology, neurology and palliative care). Prescribers/doctors from these teams	46	Observations Semi-structured interviews		Thematic analysis
Baysari et al. (2012) [73]	Australia	Teaching hospital	CPOE + basic CDS	Doctors	16	Semi-structured interviews		Thematic analysis
Baysari et al. (2014) [78]	Australia	Teaching hospital	CPOE + CDS	Doctors	74 (in qualitative components)	Observations Semi-structured interviews		Thematic analysis
Baysari et al. (2017) [92]	Australia	Teaching hospital	CPOE + CDS	Doctors	11 (in qualitative component)	Semi-structured interviews		General inductive approach
Baysari et al. (2018) [61]	Australia	Acute pediatric tertiary hospital	CPOE + CDS	Doctors Nurses	114	Semi-structured interviews	Extended Technology Acceptance Model (e-TAM)	Thematic analysis
Botta & Cutler (2014) [54]	United States	17 hospital systems, representing 144 individual acute-care hospitals	EHR-based CPOE + CDS	Chief Information Officers EHR development executives (vendors)	25	Semi-structured interviews		Thematic analysis

Paper	Country	Setting	System type	Participants/user groups	Number of participants	Data collection method	Theoretical framework	Data analysis method
Burgin et al. (2012) [98]	United Kingdom	Large teaching hospital	EHR + ePrescribing	Pharmacists of all levels of seniority	20	Semi-structured focus groups	Actor Network Theory (ANT) and Engestrom's concept of human activity system from Expansive Learning Theory	Thematic analysis
Burgin et al. (2014) [97]	United Kingdom	Large teaching hospital	EHR + ePrescribing	Pharmacists of all levels of seniority	20	Semi-structured focus groups	Actor Network Theory (ANT) and Engestrom's concept of human activity system from Expansive Learning Theory	Thematic analysis
Campbell et al. (2007) [90]	United States	Five hospitals, representing different organizational types (e.g., tertiary teaching vs. private community) using either commercially or self developed CPOE systems	CPOE + CDS	Hospital administrators<br>Physicians<br>Nurses<br>Pharmacists<br>Lab workers<br>Medical records specialists<br>Information technology leaders	126	Observations<br>Semi-structured oral history interviews	Concept of overdependence on technology, drawing on Rogers' Diffusion of Innovations	Grounded theory
Campbell et al. (2009) [85]	United States	Five hospitals across three organisations (including 2 large academic general hospitals, 2 community hospitals and 1 acute care county teaching hospital)	CPOE + CDS	Hospital administrators<br>Physicians<br>Nurses<br>Pharmacists<br>Other clinical personnel<br>IT staff	126	Observations<br>Semi-structured oral history interviews		Grounded theory

Paper	Country	Setting	System type	Participants/user groups	Number of participants	Data collection method	Theoretical framework	Data analysis method
Carpenter & Gorman (2001) [83]	United States	Four hospitals, with differences in teaching status, geography and length of system use	CPOE + CDS	Physicians<br>Administrators<br>Information technology staff	16	Observations<br>Interviews		Grounded theory
Chow et al. (2015) [106]	Singapore	Adult tertiary hospital	Antibiotic CDS system	Junior physicians<br>Senior physicians	11	Focus groups		Framework approach
Cornford et al. (2010) [46]	United Kingdom	Thirteen hospitals, representing the implementation of 20 different systems	ePrescribing + CDS	DoctorsNursesPharmacistsSystems developersManagers	50	InterviewsSurvey		Thematic analysis
Cresswell et al. (2013a) [44]	United Kingdom	Multiple stakeholders representing 55 organisations - including acute (47), mental health (7) and community (1) based organisations	ePrescribing (+ CDS implemented or planned in 82% of represented sites)	Pharmacists<br>Managers<br>Consultants (medical)<br>Clinical Leads<br>Nurse Leads	85	Questionnaire, including a qualitative component (subsets of semi-structured and open-ended questions)		Thematic analysis
Cresswell et al. (2013b) [45]	United Kingdom	Multiple stakeholders with experience in implementing ePrescribing systems in UK hospitals	ePrescribing	Representatives from hospitals at various stages of implementation<br>System developers<br>Policy representatives<br>Patients<br>Academics	17	One-day event including a series of sequential, multi-disciplinary round-table discussions with two parallel groups considering the same topics	Self-developed and refined theoretical framework drawing on Actor-Network Theory	Thematic analysis

Paper	Country	Setting	System type	Participants/user groups	Number of participants	Data collection method	Theoretical framework	Data analysis method
Cresswell et al. (2014) [9]	United Kingdom	Two urban, acute care hospitals	CPOE + CDS	Pharmacists<br>Nurses<br>Doctors<br>Project managers<br>IT professionals	43	Document analysis<br>Semi-structured interviews<br>Observations	Self-developed and refined theoretical framework drawing on Actor-Network Theory	Thematic analysis
Cresswell et al. (2016a) [109]	United Kingdom	Multiple stakeholders with experience in implementing ePrescribing systems in UK hospitals	ePrescribing	Pharmacists<br>Prescribing software company representatives<br>Academics<br>Policy representatives<br>Industry representatives<br>Consultants<br>Pharmaceutical company representative<br>Innovation centre representative<br>Patient	21	Focus groups	Drawing on information infrastructure studies	Thematic analysis
Cresswell et al. (2016b) [62]	United Kingdom	Multiple stakeholders involved in the implementation of hospital electronic prescribing and medicines administration systems in Scotland	ePrescribing	Pharmacists<br>System suppliers<br>Antimicrobial prescribing group members<br>Policy representatives<br>Analytic company representative<br>Academic nurse<br>Patient association member<br>Oncology consultant<br>Pain consultant<br>Prescribing support unit member	21	Focus groups	Drawing on information infrastructure studies	Thematic analysis

Paper	Country	Setting	System type	Participants/user groups	Number of participants	Data collection method	Theoretical framework	Data analysis method
Cresswell et al. (2017a) [23]	United Kingdom	Six urban, acute care hospitals	ePrescribing + CDS	Hospital decision makers<br>Implementation teams<br>Information technology staff<br>Clinicians<br>Allied health professionals<br>System suppliers	195	Semi-structured interviews<br>Non-participant observations<br>Document analysis<br>Expert round-table discussions	Interpretation of findings drew on the concept of 'information infrastructures'	Thematic analysis
Cresswell et al. (2017b) [22]	United Kingdom	Five acute care hospitals, at various stages of implementation	ePrescribing systems with differing functionalities	Managers (information technology and clinical implementation team members) involved in implementing ePrescribingUsers (including doctors, nurses, pharmacists, allied health professionals and pharmacy technicians varying in levels of seniority and experience with ePrescribing system use)	173	InterviewsNon-participant observationDocument analysis	Interpretation of findings drew on the Theory of Workarounds	Thematic analysis
Cresswell et al (2017c) [59]	United Kingdom	Six hospitals	CPOE + CDS	Clinical users (physicians, nurses, pharmacists, and other allied health professionals)<br>Support staff (technicians)<br>System vendors<br>Managers (implementation teams, information technology specialists) of varying levels of seniority	197	Semi-structured interviews<br>Non-participant observations Document analysis		Thematic analysis

Paper	Country	Setting	System type	Participants/user groups	Number of participants	Data collection method	Theoretical framework	Data analysis method
Davidson & Chismar (2007) [47]	United States	One urban, acute care hospital	CPOE	Physicians from different specialties (cardiology, general surgery, internal medicine, orthopedics, oncology)<br>Nurses and nurse administrators<br>Unit secretaries<br>Pharmacists<br>Laboratory supervisors<br>Respiratory therapists<br>Health-care analysts<br>Information Systems staff members<br>Chief Information Officer	32	Semi-structured interviews	Barley's (1990) model for the alignment of technology and social structure, and Scott's (2001) institutional theory.	Theoretically informed framework (Barley, 1990) drawing on the principle of the hermeneutic circle
Debono et al. (2017) [88]	Australia	Two large urban teaching hospitals	ePrescribing + CDS	Nurses	19	In-depth interviews	Theoretical Domains Framework (TDF)	Thematic analysis
Dixon-Woods et al. (2013) [20]	United Kingdom	One large, acute care hospital	ePrescribing + CDS	Front-line staff<br>Hospital senior executives	10 +<br>190 hours of observations	Ethnographic observations<br>Informal conversations<br>Semistructured interviews<br>Document analysis		Constant comparative method
Dykstra (2002) [101]	United States	Four hospitals	CPOE	Physicians<br>Administrators<br>IT staff<br>Pharmacists<br>Nurses	87	Observation<br>Formal oral history interviews<br>Focus groups<br>Informal interviews		Grounded theory
Garfield et al (2016) [100]	United Kingdom	Two large teaching hospitals	ePrescribing	Adult inpatients<br>Carers<br>Healthcare professionals	590	Non–participant observations<br>Semi structured interviews		Thematic analysis
Griffon et al. (2017) [60]	France	One university hospital	COPE + CDS	Doctors (residents)	71	Questionnaire, including a qualitative component (free-text data)		Thematic analysis

Paper	Country	Setting	System type	Participants/user groups	Number of participants	Data collection method	Theoretical framework	Data analysis method
Hardie et al. (2017) [56]	Australia	Eight wards in an urban children's hospital	COPE + CDS	Doctors<br>Nurses	90	Semi-structured interviews		Thematic analysis
Hawkins et al. (2017) [71]	United States	One midsized hospital	CPOE	Administrative level staff (in nursing, quality, education, and pharmacy)<br>Medical staff<br>Pharmacists<br>Registered nurses	37	Unstructured interviews<br>Observations<br>Document reviews		Conventional content analysis
Holden (2010) [82]	United States	Two large tertiary hospitals	EHR + CPOE	Physicians	20	Semi-structured interviews	Theory of Planned Behavior	Thematic analysis
Holden (2011) [69]	United States	Two large tertiary hospitals	EHR + CPOE	Physicians	20	Semi-structured interviews	Human factors paradigm for patient safety and Theory of Planned Behavior	Thematic analysis
Jeon et al. (2014) [49]	Canada	One urban general hospital	CPOE	Medical oncologists<br>Oncology pharmacists<br>Oncology nurses<br>Informaticians<br>Human factors engineers<br>Patient safety researchers<br>Policymakers<br>Hospital administrators	30 (in qualitative component)	Workshop discussions		Thematic analysis
Johnson et al. (2006) [96]	United States	Two hospitals	CPOE	Internal medicine physicians	10	Think-aloud observations<br>Semi-structured questionnaire		Thematic analysis

Paper	Country	Setting	System type	Participants/user groups	Number of participants	Data collection method	Theoretical framework	Data analysis method
Jung et al. (2013) [79]	Netherlands<br>Argentina<br>Denmark<br>France<br>Austria<br>Ireland<br>Italy<br>Bulgaria<br>Greece<br>Switzerland	Thirteen hosptials	CPOE + varying levels of CDS	Physicians	1,018	Cross-sectional quantitative and qualitative questionnaire		Qualitative content analysis with inductive category development
Malato & Kim (2004) [41]	United States	Two acute care nursing units at a large acute care hospital	CPOE	Nurses	12 + 5 hours of observations	Open-ended interviews<br>Ethnographic observations		Domain analysis
McMullen et al. (2015) [70]	United States	Three hosptials	CPOE	Pharmacists	96	Ethnographic observations<br>Focus groups<br>Interviews		Thematic analysis
Mills et al. (2017) [65]	United Kingdom	One district general hospital	ePrescribing + CDS	Consultant doctors<br>Junior doctors<br>Pharmacists<br>Advanced nurse practitioners	19	Semi-structured interviews	Theoretical Domains Framework	Framework analysis
Mozaffar et al. (2016a) [21]	United Kingdom	Six urban acute care hospitals	CPOE + CDS	Implementation team members<br>Systems users - clinical staff<br>Policymakers<br>Representatives of suppliers<br>Academics<br>Patients	189	Semi-structured interviews<br>Expert roundtable discussions<br>Document analysis	Biography of Artefacts	Thematic analysis

Paper	Country	Setting	System type	Participants/user groups	Number of participants	Data collection method	Theoretical framework	Data analysis method
Mozaffar et al. (2016b) [64]	United Kingdom	Vendors and adopters from multiple hospitals in England	CPOE + CDS	Representatives from 4 vendors (project managers)<br>Representatives from 6 hospitals (project management team members, such as doctors and pharmacists)	11 + 21 hours of observation	Semi-structured interviews<br>Observation	Informed by studies of the evolution of technology fields	Thematic analysis
Mozaffar et al. (2017) [14]	United Kingdom	Six urban acute care hospitals	ePrescribing + CDS	Clinical users (consultants, junior doctors, nurses, pharmacists and other health professionals)<br>Decision makers and managers (implementation teams, information technology specialists)<br>Technical support staff<br>System suppliers	163 + 35 hours of observations	Semi-structured interviews<br>Observations<br>Document analysis	Biography of Artefacts	Thematic analysis
Niazkhani et al. (2008) [102]	Netherlands	One academic urban hospital	CPOE	Nurses<br>Physicians<br>Pharmacists<br>Pharmacy technicians	23	Semi-structured interviews<br>Document analysis	Self-developed conceptual framework for inter-professional workflow in the medication process	Thematic analysis
Niazkhani et al. (2010a) [94]	Netherlands	One academic urban hospital	CPOE	Non-surgical: physicians, residents, head nurses and practicing nurses<br>Surgical: surgeons, surgery residents, head nurses and practicing nurses	22	Semi-structured interviews<br>Observations		Thematic analysis

Paper	Country	Setting	System type	Participants/user groups	Number of participants	Data collection method	Theoretical framework	Data analysis method
Niazkhani et al. (2010b) [103]	Netherlands	One academic urban hospital	CPOE	Nurses<br>Physicians<br>Pharmacists<br>Pharmacy technicians	23	Semi-structured interviews<br>Document analysis	Self-developed conceptual framework for inter-professional workflow in the medication process	Thematic analysis
Niazkhani et al. (2011) [84]	Netherlands	One academic urban hospital	CPOE	Nurses<br>Physicians<br>Pharmacists<br>Pharmacy technicians	21	Semi-structured interviews<br>Document analysis		Thematic analysis
Nies & Pelayo (2010) [87]	France	One large academic hospital	EHR + CPOE	Physicians	10 + 20 observations	Semi-structured interviewsParticipant observations	Human Factors Engineering approach	Human Factors' Activity Analysis
O'Grady et al. (2006) [72]	United Kingdom	One general surgery ward	ePrescribing	Inpatients	20	Interviews		Thematic analysis
Omar et al. (2017) [75]	Sweden	Pediatric department of university hospital	ePrescribing + CDS	Pediatricians<br>Resident physicians<br>Pediatric surgeons<br>Neonatologists	Not reported	Semi-structured interviews	Extended Technology Acceptance Model (TAM2)	Not reported
Pelayo et al. (2010) [99]	France	Three hospitals	CPOE	Doctors<br>Nurses	44 + 55 observations (3-8 hours)	Naturalistic observations<br>Interviews		Not reported
Pirnejad et al. (2008) [104]	Netherlands	Six internal medicine wards at an urban academic hospital	CPOE + CDS	Nurses<br>Physicians	15 (in qualitative component)	Semi-structured interviews	Theoretical framework drawing on the concepts of articulation and interoperability	Thematic analysis

Paper	Country	Setting	System type	Participants/user groups	Number of participants	Data collection method	Theoretical framework	Data analysis method
							in collaborative work	
Pirnejad et al. (2009) [74]	Netherlands	Six internal medicine wards at an urban academic hospital	CPOE + CDS	Nurses<br>Physicians	15 (in qualitative component)	Semi-structured interviews	Theoretical framework drawing on the concepts of articulation and interoperability in collaborative work	Thematic analysis
Pirnejad et al. (2011) [50]	Netherlands	Six internal medicine wards at an urban academic hospital	CPOE + CDS	Physicians<br>Nurses<br>Project leaders	12	Semi-structured interviews		Thematic analysis
Puaar and Franklin (2018) [63]	United Kingdom	One large teaching hospital	ePrescribing + CDS	Doctors<br>Nurses	25	Semi-structured interviews	Framework based on Reason's accident causation model	Thematic analysis
Redwood et al. (2013) [77]	United Kingdom	One large teaching hospital	ePrescribing + CDS	Junior doctors	19 (in qualitative component)	Focus groups<br>Semi-structured interviews		Constant comparative method
Riccioli et al. (2011) [68]	Italy	One internal medicine ward	ePrescribing	Head physician<br>Head nurse<br>Physicians	25	Semi-structured interviews<br>In-depth questionnaire		Not reported
Santucci et al (2016) [76]	Australia	One general/neurological intensive care unit (ICU) at a teaching hospital	ePrescribing + CDS	Senior doctors<br>Junior doctors	20	Observations<br>Interviews		Thematic analysis

**Paper**	**Country**	**Setting**	**System type**	**Participants/user groups**	**Number of participants**	**Data collection method**	**Theoretical framework**	**Data analysis method**
Savage et al. (2010) [89]	United Kingdom	One district general hospital	ePrescribing + CDS	Doctors<br>Nurses<br>Pharmacists<br>Pharmacy technicians<br>Adult in-patients	45 (in qualitative component)	Interviews		Thematic analysis
Shemilt et al. (2017) [93]	United Kingdom	65 general acute hospitals in England	ePrescribing	Chief pharmacists (or their nominated representatives)	65	Semi-structured telephone interviews		Grounded theory
Simon et al. (2013) [19]	United States	Five community hospitals	CPOE + CDS	Physicians<br>Nurses<br>Pharmacists	24 + 8 hours of observation per site	Structured observations<br>In-depth, semi-structured interviews		Content analysis
Sittig et al. (2005) [107]	United States	Five hospitals across four organisations, with differences in teaching status, geography and length of system use	CPOE	Physicians<br>Administrators<br>Information technology staff<br>Pharmacists<br>Nurses	74	Participant observation<br>Oral history interviews<br>Focus groups	Storm and Storm's (1987) taxonomy of emotions	Thematic analysis
Tschannen et al (2011) [67]	United States	Two inpatient units (adult medical and general pediatric) in a large tertiary-care hospital	CPOE	Nurses	86	Semi-structured interviews	DeLone and McLean's (2003) Information Systems Success Model	Content analysis
van der Sijs et al. (2008) [91]	Netherlands	One urban general hospital	CPOE + CDS	Physicians<br>Pharmacists	24 (in qualitative component)	Interviews		Thematic analysis
Wentzer et al. (2007) [86]	Denmark	Two internal medicine wards	CPOE	Physicians<br>Nurses	6 + 48 hours of observation	Observations<br>Semi-structured interviews<br>Document analysis	Socio-technical approach	Not reported
Wong et al. (2012) [105]	Canada	Fve urban teaching hospitals	CPOE + CDS	Internal medicine residents<br>Attending physicians	17	Semi-structured interviews		Grounded theory

Paper	Country	Setting	System type	Participants/user groups	Number of participants	Data collection method	Theoretical framework	Data analysis method
Yang et al. (2012) [66]	Singapore	One public hospital	CPOE	Adult physiciansAdult nursesIT staffPediatric pharmacistPediatric physiciansPediatric nurses	30	InterviewsDocument analysis	A combination of 'accommodation to misfit' (Gasser, 1986) and 'information system evolution' (McGannand Lyytinen, 2008) perspectives	Thematic analysis and process framework (Langley, 1999) development
Zhou et al. (2011) [108]	United States	One internal medicine unit at a large teaching hospital	CPOE	Doctors<br>Residents on monthly rotation<br>Nurses	103	Observations<br>Document analysis<br>Semi-structured interviews	Symbolic interactionism perspective, focusing on the use of the concepts of 'constant negotiation', 'boundary object' and 'assemblage'	Thematic analysis
