# Supplementary material for: How do stakeholders experience the adoption of electronic prescribing systems in hospitals? A systematic review and thematic synthesis of qualitative studies
Source: BMJ Qual Saf. 2019 Jul 29;28(12):1021–31. doi: 10.1136/bmjqs-2018-009082 (PMC6934241; doi:10.1136/bmjqs-2018-009082)
Supplement: Supplementary data [file bmjqs-2018-009082supp005.pdf]

Supplementary Table 4. Quality assessment of included studies (n=79)

Paper	Approach and design																				Data collection																Data analysis and findings												Other	
	1. Was the research problem and/or research question clearly reported/defined?				2. Was there a clear statement of the aims and/or objectives of the research?				3. Was a qualitative methodology appropriate?				4. Was the research design appropriate to address the aims of the research?				5. Was the sampling and recruitment strategy clearly defined and justified?				6. Was the method of data collection well described?				7. Were any techniques to enhance trustworthiness used?				8. Has the relationship between researchers and participants been adequately considered?				9. Have ethical issues been taken into consideration?				10. Was the data analysis/interpretation process well described and justified?				11. Was there a clear statement of findings?				12. Are the analysis and findings credible?				13. Was any conflict of interest reported?	
	Yes	Partially	No	Unclear	Yes	Partially	No	Unclear	Yes	Partially	No	Unclear	Yes	Partially	No	Unclear	Yes	Partially	No	Unclear	Yes	Partially	No	Unclear	Yes	Partially	No	Unclear	Yes	Partially	No	Unclear	Yes	Partially	No	Unclear	Yes	Partially	No	Unclear	Yes	Partially	No	Unclear	Yes	Partially	No	Unclear	Yes	No
Aarts et al. (2004) [55]	●				●				●				●				●				●					●					●			●					●		●					●				●
Abraham et al. (2018) [95]	●				●					●			●				●				●				●					●			●				●				●				●					●
Abramson et al. (2012) [18]	●				●				●				●				●				●				●						●		●				●				●				●					●
Abramson et al. (2016) [81]			●		●				●				●				●				●				●					●			●				●				●				●					●
Ash et al. (1999) [24]	●				●				●				●					●			●				●					●				●			●				●				●					●
Ash et al. (2000) [48]	●				●				●				●					●			●				●					●				●			●				●					●				●
Ash et al. (2001a) [51]			●				●		●							●		●			●				●						●			●			●					●				●				●
Ash et al. (2001b) [57]		●			●				●				●					●			●					●					●			●				●				●				●				●
Ash et al. (2003a) [43]			●		●				●				●					●			●				●						●			●			●				●				●					●

Supplementary Table 4. Quality assessment of included studies (n=79)

Approach and design

Paper	1. Was the research problem and/or research question clearly reported/defined?				2. Was there a clear statement of the aims and/or objectives of the research?				3. Was a qualitative methodology appropriate?				4. Was the research design appropriate to address the aims of the research?				5. Was the sampling and recruitment strategy clearly defined and justified?			
	Yes	Partially	No	Unclear	Yes	Partially	No	Unclear	Yes	Partially	No	Unclear	Yes	Partially	No	Unclear	Yes	Partially	No	Unclear
Ash et al. (2003b) [42]			●		●				●				●					●		
Ash et al. (2003c) [58]	●				●				●				●					●		
Ash et al. (2005) [52]	●					●			●							●		●		
Ash et al. (2012) [53]			●		●					●			●					●		
Barber et al. (2007) [16]			●		●				●				●				●			
Baysari et al. (2011) [80]			●		●				●				●				●			
Baysari et al. (2012) [73]			●		●				●				●					●		
Baysari et al. (2014) [78]		●			●					●			●					●		
Baysari et al. (2017) [92]			●		●					●			●				●			
Baysari et al. (2018) [61]		●			●				●				●				●			
Botta & Cutler			●		●					●			●				●			

Data collection

Paper	6. Was the method of data collection well described?				7. Were any techniques to enhance trustworthiness used?				8. Has the relationship between researchers and participants been adequately considered?				9. Have ethical issues been taken into consideration?			
	Yes	Partially	No	Unclear	Yes	Partially	No	Unclear	Yes	Partially	No	Unclear	Yes	Partially	No	Unclear
Ash et al. (2003b) [42]	●				●						●			●		
Ash et al. (2003c) [58]	●				●						●			●		
Ash et al. (2005) [52]	●				●						●			●		
Ash et al. (2012) [53]	●						●				●			●		
Barber et al. (2007) [16]	●				●						●			●		
Baysari et al. (2011) [80]	●					●					●			●		
Baysari et al. (2012) [73]	●					●					●			●		
Baysari et al. (2014) [78]		●					●				●			●		
Baysari et al. (2017) [92]	●				●						●			●		
Baysari et al. (2018) [61]	●				●					●			●			
Botta & Cutler	●					●					●			●		

Data analysis and findings

Paper	10. Was the data analysis/interpretation process well described and justified?				11. Was there a clear statement of findings?				12. Are the analysis and findings credible?			
	Yes	Partially	No	Unclear	Yes	Partially	No	Unclear	Yes	Partially	No	Unclear
Ash et al. (2003b) [42]	●				●				●			
Ash et al. (2003c) [58]	●				●				●			
Ash et al. (2005) [52]	●				●				●			
Ash et al. (2012) [53]		●			●					●		
Barber et al. (2007) [16]	●				●				●			
Baysari et al. (2011) [80]		●			●				●			
Baysari et al. (2012) [73]		●			●				●			
Baysari et al. (2014) [78]			●		●				●			
Baysari et al. (2017) [92]	●				●				●			
Baysari et al. (2018) [61]	●				●				●			
Botta & Cutler			●		●				●			

Other

Paper	13. Was any conflict of interest reported?	
	Yes	No
Ash et al. (2003b) [42]		●
Ash et al. (2003c) [58]		●
Ash et al. (2005) [52]		●
Ash et al. (2012) [53]		●
Barber et al. (2007) [16]		●
Baysari et al. (2011) [80]		●
Baysari et al. (2012) [73]		●
Baysari et al. (2014) [78]		●
Baysari et al. (2017) [92]		●
Baysari et al. (2018) [61]		●
Botta & Cutler		●

Supplementary Table 4. Quality assessment of included studies (n=79)

Paper	Approach and design																				Data collection																Data analysis and findings												Other	
	1. Was the research problem and/or research question clearly reported/defined?				2. Was there a clear statement of the aims and/or objectives of the research?				3. Was a qualitative methodology appropriate?				4. Was the research design appropriate to address the aims of the research?				5. Was the sampling and recruitment strategy clearly defined and justified?				6. Was the method of data collection well described?				7. Were any techniques to enhance trustworthiness used?				8. Has the relationship between researchers and participants been adequately considered?				9. Have ethical issues been taken into consideration?				10. Was the data analysis/interpretation process well described and justified?				11. Was there a clear statement of findings?				12. Are the analysis and findings credible?				13. Was any conflict of interest reported?	
	Yes	Partially	No	Unclear	Yes	Partially	No	Unclear	Yes	Partially	No	Unclear	Yes	Partially	No	Unclear	Yes	Partially	No	Unclear	Yes	Partially	No	Unclear	Yes	Partially	No	Unclear	Yes	Partially	No	Unclear	Yes	Partially	No	Unclear	Yes	Partially	No	Unclear	Yes	Partially	No	Unclear	Yes	Partially	No	Unclear	Yes	No
(2014) [54]																																																		
Burgin et al. (2012) [98]	●				●				●				●				●				●						●				●			●			●				●				●					●
Burgin et al. (2014) [97]			●		●				●				●				●				●						●		●					●			●				●				●					●
Campbell et al. (2007) [90]	●						●		●				●				●					●					●				●					●		●			●				●					●
Campbell et al. (2009) [85]			●			●			●				●						●			●						●			●			●			●				●				●					●
Carpenter & Gorman (2001) [83]	●						●		●							●				●			●					●			●					●			●		●				●					●
Chow et al. (2015) [106]			●		●					●			●						●		●					●				●				●					●			●				●				●
Cornford et al.			●		●				●				●						●				●				●				●				●				●		●							●		●

Supplementary Table 4. Quality assessment of included studies (n=79)

Paper	Approach and design																				Data collection																Data analysis and findings												Other	
	1. Was the research problem and/or research question clearly reported/defined?				2. Was there a clear statement of the aims and/or objectives of the research?				3. Was a qualitative methodology appropriate?				4. Was the research design appropriate to address the aims of the research?				5. Was the sampling and recruitment strategy clearly defined and justified?				6. Was the method of data collection well described?				7. Were any techniques to enhance trustworthiness used?				8. Has the relationship between researchers and participants been adequately considered?				9. Have ethical issues been taken into consideration?				10. Was the data analysis/interpretation process well described and justified?				11. Was there a clear statement of findings?				12. Are the analysis and findings credible?				13. Was any conflict of interest reported?	
	Yes	Partially	No	Unclear	Yes	Partially	No	Unclear	Yes	Partially	No	Unclear	Yes	Partially	No	Unclear	Yes	Partially	No	Unclear	Yes	Partially	No	Unclear	Yes	Partially	No	Unclear	Yes	Partially	No	Unclear	Yes	Partially	No	Unclear	Yes	Partially	No	Unclear	Yes	Partially	No	Unclear	Yes	Partially	No	Unclear	Yes	No
(2010) [46]																																																		
Cresswell et al. (2013a) [44]			●		●					●			●				●				●						●				●		●				●				●				●					●
Cresswell et al. (2013b) [45]			●		●				●				●				●				●						●				●		●				●				●				●					●
Cresswell et al. (2014) [9]	●					●			●				●				●				●				●							●	●				●				●				●					●
Cresswell et al. (2016a) [109]		●			●				●				●				●				●				●					●			●				●				●				●					●
Cresswell et al. (2016b) [62]		●			●				●				●				●				●				●					●			●				●				●				●					●
Cresswell et al. (2017a) [23]			●		●				●				●				●				●							●			●		●				●				●				●					●
Cresswell et al. (2017b) [22]			●		●				●				●				●				●				●						●		●				●				●				●					●

Supplementary Table 4. Quality assessment of included studies (n=79)

Paper	Approach and design																			
	1. Was the research problem and/or research question clearly reported/defined?				2. Was there a clear statement of the aims and/or objectives of the research?				3. Was a qualitative methodology appropriate?				4. Was the research design appropriate to address the aims of the research?				5. Was the sampling and recruitment strategy clearly defined and justified?			
	Yes	Partially	No	Unclear	Yes	Partially	No	Unclear	Yes	Partially	No	Unclear	Yes	Partially	No	Unclear	Yes	Partially	No	Unclear
Cresswell et al (2017c) [59]			●		●				●				●				●			
Davidson & Chismar (2007) [47]			●		●				●				●				●			
Debono et al. (2017) [88]			●		●				●				●				●			
Dixon-Woods et al. (2013) [20]	●				●				●				●				●			
Dykstra (2002) [101]			●			●			●				●							●
Garfield et al (2016) [100]	●				●				●				●				●			
Griffon et al. (2017) [60]		●			●					●			●				●			
Hardie et al. (2017) [56]			●		●				●				●					●		

Paper	Data collection															
	6. Was the method of data collection well described?				7. Were any techniques to enhance trustworthiness used?				8. Has the relationship between researchers and participants been adequately considered?				9. Have ethical issues been taken into consideration?			
	Yes	Partially	No	Unclear	Yes	Partially	No	Unclear	Yes	Partially	No	Unclear	Yes	Partially	No	Unclear
Cresswell et al (2017c) [59]	●							●			●		●			
Davidson & Chismar (2007) [47]	●							●		●					●	
Debono et al. (2017) [88]	●				●						●		●			
Dixon-Woods et al. (2013) [20]	●				●							●	●			
Dykstra (2002) [101]			●				●				●				●	
Garfield et al (2016) [100]	●				●				●				●			
Griffon et al. (2017) [60]	●				●						●			●		
Hardie et al. (2017) [56]		●			●						●			●		

Paper	Data analysis and findings												Other	
	10. Was the data analysis/interpretation process well described and justified?				11. Was there a clear statement of findings?				12. Are the analysis and findings credible?				13. Was any conflict of interest reported?	
	Yes	Partially	No	Unclear	Yes	Partially	No	Unclear	Yes	Partially	No	Unclear	Yes	No
Cresswell et al (2017c) [59]	●				●				●					●
Davidson & Chismar (2007) [47]	●				●				●					●
Debono et al. (2017) [88]	●				●				●					●
Dixon-Woods et al. (2013) [20]	●				●				●					●
Dykstra (2002) [101]		●				●				●				●
Garfield et al (2016) [100]	●				●				●					●
Griffon et al. (2017) [60]		●			●					●				●
Hardie et al. (2017) [56]		●			●				●					●

Supplementary Table 4. Quality assessment of included studies (n=79)

Paper	Approach and design																			
	1. Was the research problem and/or research question clearly reported/defined?				2. Was there a clear statement of the aims and/or objectives of the research?				3. Was a qualitative methodology appropriate?				4. Was the research design appropriate to address the aims of the research?				5. Was the sampling and recruitment strategy clearly defined and justified?			
	Yes	Partially	No	Unclear	Yes	Partially	No	Unclear	Yes	Partially	No	Unclear	Yes	Partially	No	Unclear	Yes	Partially	No	Unclear
Hawkins et al. (2017) [71]	●				●				●				●				●			
Holden (2010) [82]	●				●				●				●				●			
Holden (2011) [69]	●				●				●				●				●			
Jeon et al. (2014) [49]	●				●					●				●				●		
Johnson et al. (2006) [96]	●				●				●					●					●	
Jung et al. (2013) [79]	●				●				●						●		●			
Malato & Kim (2004) [41]	●				●				●					●				●		

Paper	Data collection															
	6. Was the method of data collection well described?				7. Were any techniques to enhance trustworthiness used?				8. Has the relationship between researchers and participants been adequately considered?				9. Have ethical issues been taken into consideration?			
	Yes	Partially	No	Unclear	Yes	Partially	No	Unclear	Yes	Partially	No	Unclear	Yes	Partially	No	Unclear
Hawkins et al. (2017) [71]	●				●					●				●		
Holden (2010) [82]	●				●				●					●		
Holden (2011) [69]	●				●				●					●		
Jeon et al. (2014) [49]		●				●					●					●
Johnson et al. (2006) [96]		●				●					●			●		
Jung et al. (2013) [79]	●							●			●			●		
Malato & Kim (2004) [41]		●				●					●			●		

Paper	Data analysis and findings												Other	
	10. Was the data analysis/interpretation process well described and justified?				11. Was there a clear statement of findings?				12. Are the analysis and findings credible?				13. Was any conflict of interest reported?	
	Yes	Partially	No	Unclear	Yes	Partially	No	Unclear	Yes	Partially	No	Unclear	Yes	No
Hawkins et al. (2017) [71]	●				●				●					●
Holden (2010) [82]	●				●				●					●
Holden (2011) [69]	●				●				●					●
Jeon et al. (2014) [49]		●			●					●				●
Johnson et al. (2006) [96]			●			●			●					●
Jung et al. (2013) [79]		●			●					●				●
Malato & Kim (2004) [41]	●				●				●					●

Supplementary Table 4. Quality assessment of included studies (n=79)

Paper	Approach and design																				Data collection																Data analysis and findings												Other	
	1. Was the research problem and/or research question clearly reported/defined?				2. Was there a clear statement of the aims and/or objectives of the research?				3. Was a qualitative methodology appropriate?				4. Was the research design appropriate to address the aims of the research?				5. Was the sampling and recruitment strategy clearly defined and justified?				6. Was the method of data collection well described?				7. Were any techniques to enhance trustworthiness used?				8. Has the relationship between researchers and participants been adequately considered?				9. Have ethical issues been taken into consideration?				10. Was the data analysis/interpretation process well described and justified?				11. Was there a clear statement of findings?				12. Are the analysis and findings credible?				13. Was any conflict of interest reported?	
	Yes	Partially	No	Unclear	Yes	Partially	No	Unclear	Yes	Partially	No	Unclear	Yes	Partially	No	Unclear	Yes	Partially	No	Unclear	Yes	Partially	No	Unclear	Yes	Partially	No	Unclear	Yes	Partially	No	Unclear	Yes	Partially	No	Unclear	Yes	Partially	No	Unclear	Yes	Partially	No	Unclear	Yes	Partially	No	Unclear	Yes	No
McMullen et al. (2015) [70]	●				●				●					●					●			●						●			●			●				●				●				●			●	
Mills et al. (2017) [65]	●				●				●				●				●				●				●					●			●				●				●				●					●
Mozaffar et al. (2016a) [21]	●				●				●				●				●				●				●					●				●			●				●				●					●
Mozaffar et al. (2016b) [64]	●				●				●				●				●				●				●				●				●				●				●				●					●
Mozaffar et al. (2017) [14]	●				●				●				●				●				●				●				●					●			●				●				●					●
Niazkhani et al. (2008) [102]	●				●				●					●				●				●				●					●					●		●			●				●					●
Niazkhani et al. (2010a) [94]	●				●				●				●				●				●					●					●			●			●				●				●					●
Niazkhani et al. (2010b) [103]	●				●				●					●				●			●					●				●						●		●			●				●					●

Supplementary Table 4. Quality assessment of included studies (n=79)

Paper	Approach and design																				Data collection																Data analysis and findings												Other	
	1. Was the research problem and/or research question clearly reported/defined?				2. Was there a clear statement of the aims and/or objectives of the research?				3. Was a qualitative methodology appropriate?				4. Was the research design appropriate to address the aims of the research?				5. Was the sampling and recruitment strategy clearly defined and justified?				6. Was the method of data collection well described?				7. Were any techniques to enhance trustworthiness used?				8. Has the relationship between researchers and participants been adequately considered?				9. Have ethical issues been taken into consideration?				10. Was the data analysis/interpretation process well described and justified?				11. Was there a clear statement of findings?				12. Are the analysis and findings credible?				13. Was any conflict of interest reported?	
	Yes	Partially	No	Unclear	Yes	Partially	No	Unclear	Yes	Partially	No	Unclear	Yes	Partially	No	Unclear	Yes	Partially	No	Unclear	Yes	Partially	No	Unclear	Yes	Partially	No	Unclear	Yes	Partially	No	Unclear	Yes	Partially	No	Unclear	Yes	Partially	No	Unclear	Yes	Partially	No	Unclear	Yes	Partially	No	Unclear	Yes	No
Niazkhani et al. (2011) [84]		●			●				●					●				●			●				●					●						●	●				●				●					●
Nies & Pelayo (2010) [87]		●			●				●						●				●			●					●				●					●			●			●					●		●	
O'Grady et al. (2006) [72]			●		●				●				●					●				●					●				●			●					●			●				●				●
Omar et al. (2017) [75]			●			●			●				●						●				●					●			●				●				●		●					●				●
Pelayo et al. (2010) [99]	●				●					●			●					●				●					●				●					●			●			●				●				●
Pirnejad et al. (2008) [104]	●				●					●			●						●		●				●						●			●				●			●				●				●	
Pirnejad et al. (2009) [74]	●				●				●				●				●				●				●						●			●			●				●				●					●
Pirnejad et al. (2011) [50]	●					●			●				●					●				●						●			●				●			●			●					●				●

Supplementary Table 4. Quality assessment of included studies (n=79)

Paper	Approach and design																				Data collection																Data analysis and findings												Other	
	1. Was the research problem and/or research question clearly reported/defined?				2. Was there a clear statement of the aims and/or objectives of the research?				3. Was a qualitative methodology appropriate?				4. Was the research design appropriate to address the aims of the research?				5. Was the sampling and recruitment strategy clearly defined and justified?				6. Was the method of data collection well described?				7. Were any techniques to enhance trustworthiness used?				8. Has the relationship between researchers and participants been adequately considered?				9. Have ethical issues been taken into consideration?				10. Was the data analysis/interpretation process well described and justified?				11. Was there a clear statement of findings?				12. Are the analysis and findings credible?				13. Was any conflict of interest reported?	
	Yes	Partially	No	Unclear	Yes	Partially	No	Unclear	Yes	Partially	No	Unclear	Yes	Partially	No	Unclear	Yes	Partially	No	Unclear	Yes	Partially	No	Unclear	Yes	Partially	No	Unclear	Yes	Partially	No	Unclear	Yes	Partially	No	Unclear	Yes	Partially	No	Unclear	Yes	Partially	No	Unclear	Yes	Partially	No	Unclear	Yes	No
Puaar and Franklin (2018) [63]		●			●				●				●				●				●				●				●				●				●				●				●				●	
Redwood et al. (2013) [77]		●			●					●			●				●				●				●				●				●				●				●				●					●
Riccioli et al. (2011) [68]			●		●					●			●					●				●						●			●					●			●			●				●				●
Santucci et al (2016) [76]		●			●				●				●				●				●				●						●		●					●			●				●					●
Savage et al. (2010) [89]			●		●					●			●				●					●			●						●		●					●			●				●					●
Shemilt et al. (2017) [93]		●			●				●					●			●					●						●			●			●				●			●					●				●
Simon et al. (2013) [19]		●			●				●				●				●				●				●				●				●				●				●				●					●
Sittig et al. (2005) [107]		●			●				●				●					●				●						●			●				●			●			●					●				●
Tschannen et al			●		●					●			●					●			●						●				●		●					●			●					●				●

Supplementary Table 4. Quality assessment of included studies (n=79)

Paper	Approach and design																				Data collection																Data analysis and findings												Other	
	1. Was the research problem and/or research question clearly reported/defined?				2. Was there a clear statement of the aims and/or objectives of the research?				3. Was a qualitative methodology appropriate?				4. Was the research design appropriate to address the aims of the research?				5. Was the sampling and recruitment strategy clearly defined and justified?				6. Was the method of data collection well described?				7. Were any techniques to enhance trustworthiness used?				8. Has the relationship between researchers and participants been adequately considered?				9. Have ethical issues been taken into consideration?				10. Was the data analysis/interpretation process well described and justified?				11. Was there a clear statement of findings?				12. Are the analysis and findings credible?				13. Was any conflict of interest reported?	
	Yes	Partially	No	Unclear	Yes	Partially	No	Unclear	Yes	Partially	No	Unclear	Yes	Partially	No	Unclear	Yes	Partially	No	Unclear	Yes	Partially	No	Unclear	Yes	Partially	No	Unclear	Yes	Partially	No	Unclear	Yes	Partially	No	Unclear	Yes	Partially	No	Unclear	Yes	Partially	No	Unclear	Yes	Partially	No	Unclear	Yes	No
(2011) [67]																																																		
van der Sijs et al. (2008) [91]	●				●					●			●				●				●						●				●			●				●			●					●				●
Wentzer et al. (2007) [86]		●				●			●				●						●			●						●			●					●			●		●					●				●
Wong et al. (2012) [105]	●				●				●				●				●				●				●				●				●				●				●				●					●
Yang et al. (2012) [66]	●				●				●				●				●				●						●			●				●				●			●				●					●
Zhou et al. (2011) [108]		●			●				●				●					●			●							●		●				●					●		●					●				●
	**35**	**16**	**28**	**0**	**69**	**7**	**3**	**0**	**63**	**16**	**0**	**0**	**66**	**8**	**2**	**3**	**44**	**24**	**9**	**2**	**57**	**18**	**4**	**0**	**38**	**12**	**15**	**14**	**10**	**16**	**51**	**2**	**24**	**39**	**6**	**10**	**43**	**22**	**14**	**0**	**69**	**10**	**0**	**0**	**55**	**22**	**1**	**1**	**4**	**75**
