# Supplementary material for: How do stakeholders experience the adoption of electronic prescribing systems in hospitals? A systematic review and thematic synthesis of qualitative studies
Source: BMJ Qual Saf. 2019 Jul 29;28(12):1021–31. doi: 10.1136/bmjqs-2018-009082 (PMC6934241; doi:10.1136/bmjqs-2018-009082)
Supplement: Supplementary data [file bmjqs-2018-009082supp006.pdf]

Supplementary Table 5: Distribution of generated themes and sub-themes by included papers

Paper	Contextualising implementation and impact		Factors affecting the implementation process		Positive and negative implications of ePrescribing/CPOE systems				Mixed impacts and change processes	
	Preparing the organisation for change	*Preparing stakeholders for change*	*Factors positively impacting implementation*	*Factors negatively impacting the implementation process*	*Positive practice implications*	*Negative practice implications*	*Positive organisational implications*	*Negative organisational implications*	*Change in practice*	*Change at the organisational level*
Aarts et al. (2004) [55]			●	●		●	●	●		
Abraham et al. (2018) [95]									●	●
Abramson et al. (2012) [18]			●		●	●			●	
Abramson et al. (2016) [81]					●	●			●	●
Ash et al. (1999) [24]	●	●	●		●	●			●	
Ash et al. (2000) [48]	●	●	●	●	●	●	●		●	●
Ash et al. (2001a) [51]			●		●	●	●	●	●	●
Ash et al. (2001b) [57]			●	●						
Ash et al. (2003a) [43]		●	●	●		●	●	●	●	●
Ash et al. (2003b) [42]			●	●	●	●			●	
Ash et al. (2003c) [58]			●							
Ash et al. (2005) [52]			●		●	●	●	●	●	●
Ash et al. (2012) [53]			●							
Barber et al. (2007) [16]			●	●	●	●			●	
Baysari et al. (2011) [80]					●	●			●	
Baysari et al. (2012) [73]					●	●				

Paper	Contextualising implementation and impact		Factors affecting the implementation process		Positive and negative implications of ePrescribing/CPOE systems				Mixed impacts and change processes	
	Preparing the organisation for change	*Preparing stakeholders for change*	*Factors positively impacting implementation*	*Factors negatively impacting the implementation process*	*Positive practice implications*	*Negative practice implications*	*Positive organisational implications*	*Negative organisational implications*	*Change in practice*	*Change at the organisational level*
Baysari et al. (2014) [78]					●	●			●	
Baysari et al. (2017) [92]						●			●	●
Baysari et al. (2018) [61]				●	●	●			●	
Botta & Cutler (2014) [54]			●							
Burgin et al. (2012) [98]									●	
Burgin et al. (2014) [97]									●	
Campbell et al. (2007) [90]						●				
Campbell et al. (2009) [85]						●			●	●
Carpenter & Gorman (2001) [83]						●			●	●
Chow et al. (2015) [106]									●	
Cornford et al. (2010) [46]	●	●	●		●	●				●
Cresswell et al. (2013a) [44]	●	●	●							
Cresswell et al. (2013b) [45]	●				●		●			
Cresswell et al. (2014) [9]					●	●			●	●
Cresswell et al. (2016a) [109]										●
Cresswell et al. (2016b) [62]				●						●
Cresswell et al. (2017a) [23]								●		

Paper	Contextualising implementation and impact		Factors affecting the implementation process		Positive and negative implications of ePrescribing/CPOE systems				Mixed impacts and change processes	
	Preparing the organisation for change	*Preparing stakeholders for change*	*Factors positively impacting implementation*	*Factors negatively impacting the implementation process*	*Positive practice implications*	*Negative practice implications*	*Positive organisational implications*	*Negative organisational implications*	*Change in practice*	*Change at the organisational level*
Cresswell et al. (2017b) [22]									●	●
Cresswell et al (2017c) [59]			●							
Davidson & Chismar (2007) [47]	●	●	●		●	●			●	●
Debono et al. (2017) [88]						●			●	
Dixon-Woods et al. (2013) [20]								●		●
Dykstra (2002) [101]									●	
Garfield et al (2016) [100]									●	
Griffon et al. (2017) [60]				●						
Hardie et al. (2017) [56]			●	●						
Hawkins et al. (2017) [71]					●	●		●	●	●
Holden (2010) [82]					●	●				
Holden (2011) [69]					●	●				
Jeon et al. (2014) [49]	●	●	●		●	●		●	●	
Johnson et al. (2006) [96]									●	
Jung et al. (2013) [79]					●	●			●	
Malato & Kim (2004) [41]				●						
McMullen et al. (2015) [70]					●	●			●	

Paper	Contextualising implementation and impact		Factors affecting the implementation process		Positive and negative implications of ePrescribing/CPOE systems				Mixed impacts and change processes	
	Preparing the organisation for change	*Preparing stakeholders for change*	*Factors positively impacting implementation*	*Factors negatively impacting the implementation process*	*Positive practice implications*	*Negative practice implications*	*Positive organisational implications*	*Negative organisational implications*	*Change in practice*	*Change at the organisational level*
Mills et al. (2017) [65]					●	●			●	
Mozaffar et al. (2016a) [21]				●						
Mozaffar et al. (2016b) [64]				●						●
Mozaffar et al. (2017) [14]						●				
Niazkhani et al. (2008) [102]									●	
Niazkhani et al. (2010a) [94]									●	●
Niazkhani et al. (2010b) [103]									●	
Niazkhani et al. (2011) [84]						●			●	
Nies & Pelayo (2010) [87]						●				
O'Grady et al. (2006) [72]					●	●				
Omar et al. (2017) [75]					●	●				
Pelayo et al. (2010) [99]									●	
Pirnejad et al. (2008) [104]									●	
Pirnejad et al. (2009) [74]					●				●	
Pirnejad et al. (2011) [50]		●	●							
Puaar and Franklin (2018) [63]				●		●			●	
Redwood et al. (2013) [77]					●		●	●		

**Paper**	**Contextualising implementation and impact**		**Factors affecting the implementation process**		**Positive and negative implications of ePrescribing/CPOE systems**				**Mixed impacts and change processes**	
	Preparing the organisation for change	***Preparing stakeholders for change***	***Factors positively impacting implementation***	***Factors negatively impacting the implementation process***	***Positive practice implications***	***Negative practice implications***	***Positive organisational implications***	***Negative organisational implications***	***Change in practice***	***Change at the organisational level***
Riccioli et al. (2011) [68]					●	●				
Santucci et al (2016) [76]					●	●				
Savage et al. (2010) [89]						●				
Shemilt et al. (2017) [93]						●	●		●	●
Simon et al. (2013) [19]			●	●	●	●				
Sittig et al. (2005) [107]									●	
Tschannen et al (2011) [67]					●	●		●	●	
van der Sijs et al. (2008) [91]						●				
Wentzer et al. (2007) [86]						●		●	●	
Wong et al. (2012) [105]									●	
Yang et al. (2012) [66]					●	●		●	●	●
Zhou et al. (2011) [108]									●	
