# Supplementary material for: How do stakeholders experience the adoption of electronic prescribing systems in hospitals? A systematic review and thematic synthesis of qualitative studies
Source: BMJ Qual Saf. 2019 Jul 29;28(12):1021–31. doi: 10.1136/bmjqs-2018-009082 (PMC6934241; doi:10.1136/bmjqs-2018-009082)
Supplement: Supplementary data [file bmjqs-2018-009082supp007.pdf]

Supplementary Table 6: Example data extracts

Themes and sub-themes		Example data extracts
**Contextualising implementation and impact**	**Preparing the organisation for change**	"The community hospital has developed a large set of clinical pathways and the development process itself seems to have positive organizational impacts as specialists, nurses, and therapists work together on them. A physician said: *'the nice part about it is it's a consensus, most of us have participated in some kind of clinical pathway development in the hospital, nursing staff are involved, pharmacy people'*." (Ash et al., 2000)<br><br>"Need for better understanding of current practices and standardization: Large variations seem to exist in workflows and practices around chemotherapy ordering and administration. These variations are not currently well understood. Successful adoption of CPOE requires identifying the sources of workflow variations and eliminating those variations and practices that could be standardized or that are unnecessary. Overall, there needs to be guidance for better standardization of practices" (Jeon et al., 2014)
	**Preparing stakeholders for change**	"Due to the variety of professions using ePrescribing systems (i.e. clinicians including junior doctors, pharmacists, nurses), engaging different staff groups as early in the process as possible, preferably in relation to systems choice, was viewed as crucial, although often not realised: *'Most systems lack clinical input and yet clinicians are expected to use systems and have little choice in system chosen'*. (Participant 18, Pharmacist)" (Cresswell et al., 2013a)<br><br>"An almost universal finding from our respondents was that eP projects must be multi-disciplinary. No one professional group can carry a successful system into widespread use. If significant professional groups are missing, excluded or unenthusiastic, then this is storing up problems ahead. As one project lead said; "Undertake lots of visits and talks, if need be grovel, go everywhere and sponsor events. Do everything to build up visibility." Many of our respondents reported a desire for more clinical participation at the outset, in particular from doctors" (Cornford et al., 2010)
**Factors affecting the implementation process**	**Factors positively impacting implementation**	"Existing findings about how user engagement may contribute to successful implementation were confirmed in this work. However, there were differences between groups in their level of engagement. We found that where a system had been designed and implemented with little involvement of particular staff groups, their interests and requirements were not well catered for—with implications for their longer-term engagement and use of the system" (Cresswell et al., 2017c)<br><br>"all hospital leaders, and a majority of the physicians, pharmacists and nurses, alluded to the importance of thoughtful composition and successful functioning of committees and working groups to establish goals, timelines and policies." (Simon et al., 2013)
	**Factors negatively impacting the implementation process**	"According to respondents, *'crashes [were] recurrent'* with CPOE and its use was *'dependent on the quality of the network and devices'*. Bugs, delays in transmission of information to the care plan, and loss of prescription were identified by many respondents. These issues caused stress and irritation among residents: *'Several times, I have had to restrain myself from throwing the computer out of the window!'*" (Griffon et al., 2017)

Themes and sub-themes		Example data extracts
		"unfamiliarity with the system was perceived to be a key attribute influencing both the time with which tasks were performed and medication safety. When asked whether the CPOE system was safer or less safe than paper charts, most participants said that although the system had the potential to be safer, during week 1 it was thought to be less safe because staff were not yet proficient in using the system. A number of participants also described particular scenarios where inexperience with the CPOE system had resulted in stress and disuse of the system" (Baysari et al., 2018)
**Positive and negative implications of ePrescribing/CPOE systems**	**Positive practice implications**	"Users were pleased with many aspects of POE. Personal order sets, groups of prewritten orders, were viewed by surgical residents as helpful for straightforward situations (...) Drug interaction alerts were also viewed as helpful, as was the shorter turnaround time for delivery of medication to the floors. The ability to enter orders from any location, graphical display that can reveal temporal patterns in data, access to knowledge sources, legibility, and access to laboratory data were all cited multiple times as definite positives. Of greatest use was *'having everybody reading off the same page'*. Anyone viewing a patient record was able to see the current status of that patient's orders." (Ash et al., 1999)<br><br>"electronic prescribing functions were positively evaluated [by junior doctors] in enhancing patient safety and reducing the amount of time spent on checking safety information" (Redwood et al., 2013)
	**Negative practice implications**	"Another difficulty in performance was created by the imposition of *'additional work'* and *'an awful lot of extra steps'*. Even though the new demands could be seen as new goal-relevant performance processes—e.g., medication reconciliation: the comparison of medications used by the patient versus medications entered in the system—they were often perceived as barriers to performance or superfluous demands that slowed down physicians' work. [...] '*it's only two clicks here and a five clicks here, but when you spend all your life clicking away'*" (Holden, 2011)<br><br>"A major task change for pharmacy and laboratory departments was the need to keep their departmental system synchronized with the CPOE system.<br>[...]<br>*We know that whatever comes across to our [pharmacy] system, our system has to match the CPOE system. The CPOE system is the chart system. We also have to look at ourselves, and prevent errors from occurring.*<br>[...]<br>*The lab people need to do a lot more thinking about how the system itself works than before....There's so many things that could go wrong with an order, and so many different places where the order could be.*<br>[...]<br>Lab supervisors believed that increased workload for electronic order handling had overwhelmed cost reductions from having orders transferred into the lab system electronically." (Davidson & Chismar, 2007)
	**Positive organisational implications**	"Just prior to the interviews taking place, a national shortage of a drug occurred; one of the interviewees explained how EPMA was a benefit regarding time and efficiency in terms of implementing a switch to another available product. '*Had we been on paper (prescribing) that would have been a very time consuming bit of logistics to sort that switch out. It was done within half a day…there are just some things where you know it just makes everything so much easier'."* (Shemilt et al., 2017)

Themes and sub-themes		Example data extracts
		"Senior managers who had implemented the more sophisticated CDS system also gave examples of how systems improved organizational performance through the implementation of new guidelines and by ensuring associated adherence of users: *…there was a manufacturing problem with Tinzaparin and we had to change to a different drug. Previously we would have had to go round every single ward, tell the doctor get them to change it. And we just put a little pop up to prompt them to do that and we knew every single patient who was on the drug so the pharmacist targeted them and within one day the whole of the hospital was changed over and normally that would have taken about a month to kick in.* Hospital B, Implementation Team, Interview 14" (Cresswell et al., 2014)
	**Negative organisational implications**	"Organization related issues refer to policies and practices by the management that do not support workflow procedures. For example, management policy requires medication to be ordered by physicians before nurses can serve the medication, but this causes issues in emergency cases when doctors do not have the time to prescribe medication through EMAS and thus nurses are unable to serve the medication promptly." (Yang et al., 2012)<br><br>"at one hospital, the EPMA system had enabled the feedback of live data to frontline staff via a quality dashboard. However, this had led to more pressures on staff with the extent of the regulation negatively affecting them. *We have some extremely comprehensive quality dash boards… There is also a lot of pressure internally now both on the nurses and the medics because of course the reporting capability within the system (EPMA) means there is nowhere to hide*." (Shemilt et al., 2017)
**Mixed impacts and change processes**	**Change in practice**	"The final category of concern related to the impact CPOE had on communication processes as nurses identified concerns with communication with other healthcare team members, including physicians and pharmacy, and the resulting impact on patient care. One nurse stated, *Nurse-physician interactions are much less frequent with CPOE..."* (Tschannen et al., 2011)<br><br>"The CPOE system in our study fundamentally reorganized the existing work procedures, affecting the workflow between the three professional groups both advantageously and disadvantageously" (Niazkhani et al., 2008)
	**Change at the organisational level**	"Interviewees emphasized the system's primary purpose as improving patient safety, particularly in directly providing decision support and a graded series of alerts and warnings for averting errors. Thought by the executive team to be very successful—capturing each year an estimated 78,000 errors of varying types and severity—the ePDSS was not originally designed to do more than serve its primary function. Over time, however, a latent secondary use of the system—one that allowed scrutiny ofpractice and performance and provided the basis for action—became evident. As a consequence, the ePDSS was re-tasked so that data created as a "by-product" of its primary function could be used to monitor quality of care close to real time at very low marginal cost." (Dixon-Woods et al., 2013)

Themes and sub-themes		Example data extracts
		"CPOE can highlight ineffective implementation of policy and procedures. CPOE systems help to formalize organizational policies and procedures. In many cases, actual practice does not match this rigid "letter of the law", so the CPOE system may introduce a significant amount of extra work (perceived or real) [...] Difficulties arise when standards are hard to interpret or implement, as when one clinician initiates patient care that must be monitored by other specialists: *'Some orders [are] written by certain specialists like anesthesiologists [for] epidurals. No one wants to rewrite those orders. So how should those [orders] traverse the levels of care when the epidural catheter moves with the patient?'* In such cases, CPOE can complicate already difficult issues." (Campbell et al., 2009)
